# Is segregation encoded in urban form? An entropy-based analysis

**DOI:** 10.1371/journal.pone.0360856

**Published:** 2026-10-23

**Authors:** Vinicius M. Netto, Caio Cacholas, Camila Carvalho, Edgardo Brigatti

**Affiliations:** 1 Research Centre for Territory, Transport and Environment (CITTA), University of Porto, Porto, Portugal; 2 Graduate Programme in Architecture and Urbanism (PPGAU), Fluminense Federal University, Niterói, Brazil; 3 Space Planning, Trinity College Dublin, Dublin, Ireland; 4 Institute of Physics, Federal University of Rio de Janeiro (UFRJ), Rio de Janeiro, Brazil; Universidade Estadual de Maringa, BRAZIL

## Abstract

The footprints of residential segregation have long been documented, yet the role of urban form as both medium and manifestation of segregation remains under-specified. We investigate whether the configuration of the built fabric may encode residential segregation in its spatial structure, hypothesising that built-form entropy (BFE) regimes are associated with the spatial distribution of income groups and their local clustering in non-linear ways. We examine this by quantifying BFE through a Shannon-based measure computed from building footprints, characterising income-based distributions using the Gini index and Moran’s I, and placing both on a common spatial footing through a regular tessellation. Applying this framework to São Paulo, Latin America’s largest city, we find non-linear relationships between BFE, income, and segregation: income levels and residential clustering increase toward both extremes of the entropy spectrum, with a stronger rise at the high-entropy end. This asymmetry suggests that high-entropy urban forms are associated with distinct spatial processes of segregation, including elite enclaving and incremental development in lower-income settlements, while low-entropy forms reflect more selective occupation shaped by planning and market filtering. Overall, the findings suggest that built form is more than a neutral backdrop, functioning as both affordance and signal of segregation.

## Introduction

The urban imprint of residential segregation has been documented in multiple ways, from early mappings of class- and occupation-based divisions in the Chicago School tradition [1] to formal indices capturing unevenness, proximity, and distance-decay in group distributions (e.g., [2]). Broadly defined as the spatial clustering of socially homogeneous groups, residential segregation has recently been identified as both the most extensively studied form of segregation and the most central node within the broader topology of segregation forms [3]. The analysis of group distributions in cities dates back at least to the late nineteenth century, notably with Charles Booth’s poverty maps of London (1889) [4] and Emily Balch’s (1895) synthesis of Hull House mappings in Chicago [5]. Since Duncan and Duncan’s (1955) foundational work [6], segregation has most often been quantified using dissimilarity-type indices, including the Gini index as a measure of evenness [7] and entropy-based measures [8]. These classic indices are non-spatial, relying just on population counts rather than on the spatial organisation of the environment itself. Subsequent advances reframed residential segregation as a scale-dependent spatial process [9], prompting the development of measures that incorporate contiguity and distance – such as boundary- and weight-modified dissimilarity indices that account for shared borders and spatial proximity [10,11] (see Supporting Information for details). This trajectory reflects a broader methodological shift from descriptive mapping to non-spatial quantification and, more recently, to explicitly spatial approaches. Within this progression, Moran’s *I* emerged as a prototypical measure of spatial autocorrelation, providing a standard way to assess whether similar social groups cluster in neighbouring locations beyond what would be expected under spatial randomness. In this sense, measures such as the Gini index capture the composition of income distributions within areas, whereas Moran’s *I* captures their spatial arrangement, distinguishing unevenness from the extent to which it is expressed as local clustering.

Yet across this progression, the physical configuration of the built environment remains largely under-specified, as spatial measures continue to capture where groups are located rather than how the morphology of urban space structures their separation and interaction. Even when spatial measures incorporate contiguity and distance, they still offer limited insight into actual residential distributions and how groups interface along the permeable public realm – streets, squares, and other open spaces between buildings where everyday interactions unfold. Adjacency alone contains little information about the internal spatial structure of urban areas. In parallel, growing interest has turned to the potential roles of urban form in shaping segregation patterns. Here, it is useful to distinguish two complementary components: the street network (SN), the connective fabric of open spaces that structures movement and accessibility, and built form (BF), understood as the shape, placement, and arrangement of buildings. Although the street network has received considerable attention in residential segregation research [12–14], the role of built form has remained comparatively under-specified, often subsumed under population distributions or generic notions of proximity. A key challenge, therefore, is to move beyond abstract measures of contiguity or distance and explicitly integrate the texture of the built environment – configurations of building footprints – into the analysis of residential segregation.

Built form may matter for residential segregation insofar as its spatial characteristics relate to where social groups locate and how they interact. We examine these characteristics with the lens of order and entropy in the built fabric. Built form is represented here two-dimensionally through building footprints, in the spirit of Nolli maps, and analysed as cellular arrangements. High built-form entropy denotes greater variability in these arrangements, typically observed where buildings are positioned with little apparent spatial order. Conversely, more ordered patterns concentrate on a narrower set of configurations, allowing them to find higher frequencies, increasing predictability and reducing entropy. Recent work suggests that entropy can encode social information associated with distinct spatial cultures [15,16]. This motivates our central research question: do configurations of built form also “encode” residential segregation?

We hypothesise that variation in built-form entropy is associated with morphological conditions under which income groups cluster, as opposed to a null expectation in which the spatial distribution of built-form entropy bears no systematic relationship to income-based residential patterns. Built-form entropy differentiates urban areas in terms of spatial order and configuration, and may be implicated in how boundaries between groups are perceived and maintained. Entropy regimes may have consequences for locational residential decisions: intricate and irregular configurations tend to reduce intervisibility and through-movement, limiting access and exposure between groups, whereas more ordered configurations tend to increase legibility, predictability, and potential co-presence (cf. [17,18]). In this sense, built-form entropy may be associated with the spatial distribution of income groups across entropy regimes, as it relates to varying conditions of accessibility, visibility, and interaction that accompany patterns of spatial separation. Higher-income households may tend to locate in highly entropic areas as soft barriers to access, as intricate spatial configurations are associated with less legible and more circuitous paths for outsiders, reduced intervisibility, and constrained through-movement, while their visual complexity may also signal distinction from surrounding areas. Conversely, lower-income communities with strong social bonds may find intricate spaces supportive of local cohesion at the expense of contact with surrounding communities [19]. Another route to high entropy can arise where formal planning is weak or absent, e.g., in settings of informal urbanisation, conditions more commonly associated with lower-income groups in Brazil. In short, income groups may – consciously or not – seek, shape, or be constrained into areas characterised by specific entropy regimes. Variation in spatial order and entropy can be mobilised in locational choices, as a morphological condition through which residential segregation emerges and persists. These considerations motivate the following complementary hypotheses:

- H1: Higher built-form entropy is associated with stronger local clustering in areas where different income groups coexist.
- H2: The association between built-form entropy and residential segregation varies across income levels, with some groups concentrating in specific entropy regimes, particularly high-income groups in high-entropy areas.
- H3: The association of built-form entropy with residential segregation is non-linear, with segregation varying across entropy values.

Taken together, these hypotheses operationalise the proposed framework by articulating complementary dimensions of how built-form entropy relates to residential segregation. H1 specifies the overall direction and intensity of the association, linking higher entropy to stronger local clustering. In other words, where the physical structure of an area is more heterogeneous, people with similar incomes may be more likely to cluster locally than mix evenly. Neighbourhoods with more diverse and entropic built forms likewise tend to show stronger clustering of income groups. H2 recognises that income groups are unevenly distributed along the built-form entropy spectrum. In practice, high-income groups tend to concentrate in high-entropy areas, middle-income groups are more broadly distributed across the spectrum, and lower-income groups are also more frequently found in relatively entropic contexts. This may also result in socially homogeneous areas with low internal inequality and clustering. H3 further refines this framework by positing a non-linear relationship, in which segregation varies across the entropy spectrum rather than following a monotonic trend. Segregation does not simply increase or decrease as urban form becomes more entropic; instead, it follows different patterns across the entropy spectrum, with intermediate-entropy contexts differing systematically from both lower- and higher-entropy areas. H1 addresses high-entropy contexts in which multiple groups coexist and cluster locally, H2 addresses the concentration of income groups in certain entropy regimes, and H3 addresses the non-linearity of the relationship between built-form entropy and residential segregation.

We aim to contribute by adding built form as a missing spatial component in residential segregation research. Rather than directly testing causal mechanisms, the analysis examines systematic spatial alignments between built-form configurations and income-based residential patterns, raising questions about how urban morphology may be implicated in segregation dynamics (Fig 1). We proceed in three steps. First, we quantify randomness in the built fabric using a corrected Shannon entropy computed from building-footprint configurations. Second, we characterise income-based residential patterns using census-tract data by observing citywide gradients in average per-capita income, and, at the local scale within grid cells, analysing inequality with Gini index and quantifying clustering using Moran’s *I*. While a segregation metric is applied on the local (cell) scale, the broader income distribution of São Paulo provides an essential contextual backdrop. Third, we examine how built-form entropy relates to these local measures across the urban territory. We apply this framework to the São Paulo metropolitan area, the largest in Latin America.

**Fig 1:**
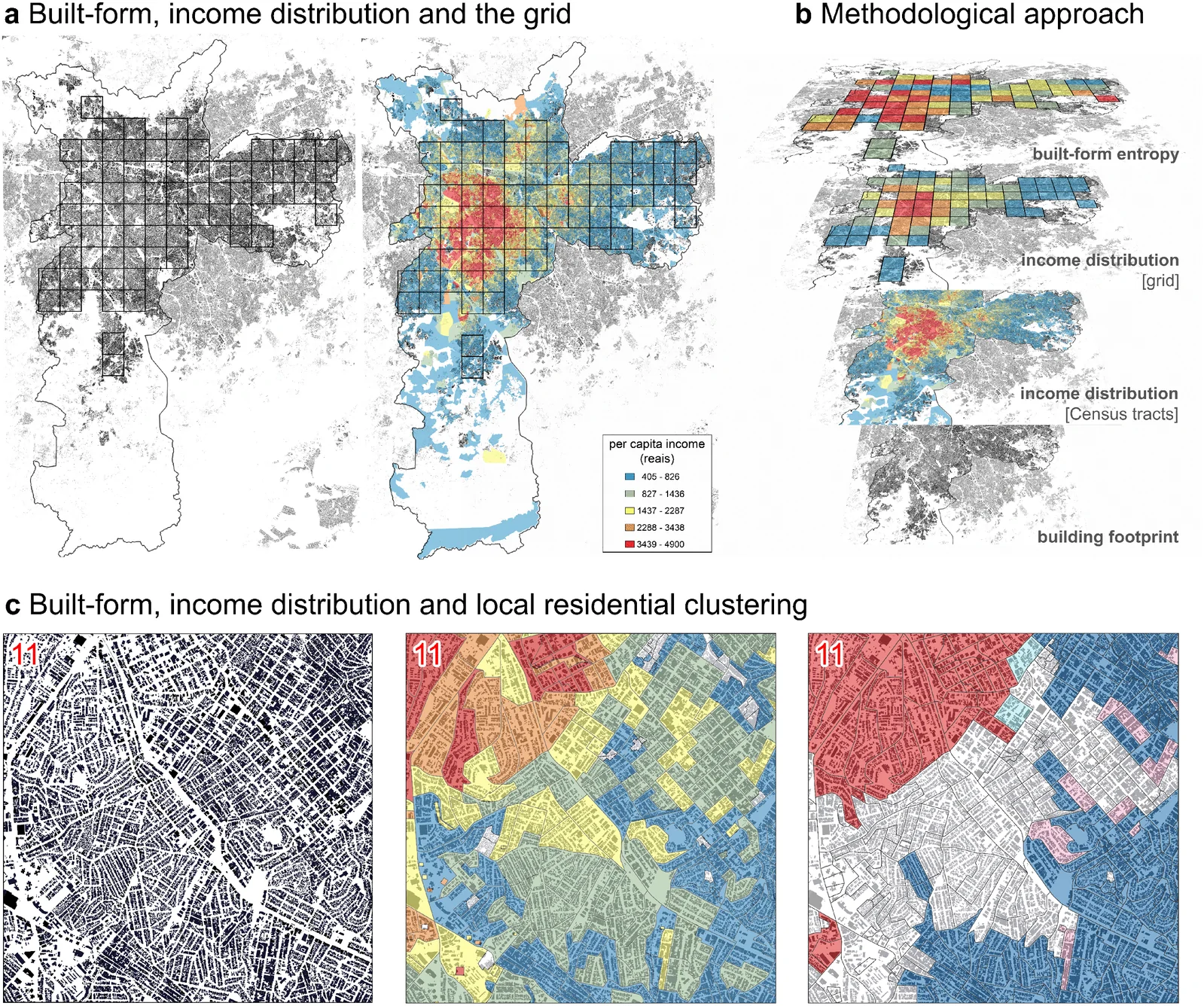
Framework linking built form and residential segregation. (a) Building footprints, tract-level average per-capita income and the regular 3×3 km grid. (b) Method schematic: overlapping the footprint and income layers to compute built-form entropy and residential-segregation metrics on the grid. (c) Grid-cell detail showing high built-form entropy, tract-level per-capita income, and Moran’s *I* (spatial autocorrelation). Here and in the following figures, the images were created by the authors using data (building footprints) from OpenStreetMap and GeoSampa (further details in the data availability statement).

## Materials and methods

We propose a measurement strategy that puts morphology and segregation on the same footing. We use a regular tessellation to place all measures on a common, equal-area spatial support. This choice serves four purposes. (i) Harmonisation: built-form entropy (from building footprints) and residential segregation (from census tracts) are reported on incompatible geometries; the grid provides a neutral carrier that avoids privileging administrative boundaries. (ii) Comparability: equal-area cells make cross-city and within-city comparisons meaningful (no size/shape biases), reducing one facet of the Modifiable Areal Unit Problem (MAUP). (iii) Statistical robustness: Built-form entropy is computed from building footprints and, therefore, requires a tessellation with sufficiently large cells to ensure reliable entropy estimates. In our study, 3×3 km cells provide a balance between spatial resolution and sample size: each cell contains enough buildings to estimate the entropy reliably and includes a substantial number of census tracts to support within-cell segregation measures. Across São Paulo, cells contain between 71 and 386 census tracts, with an average of 187 tracts per cell, providing adequate sampling for estimation of both the Gini index and Moran’s *I*. Sensitivity checks using alternative cell sizes and grid offsets indicated that smaller cells make entropy estimation impractical and clustering estimates fragile, while larger cells dilute local segregation patterns, supporting the choice of the 3×3 km tessellation as a pragmatic compromise. (iv) Reproducibility and sensitivity: a regular lattice is simple to replicate and to stress-test (offset/jittered grids, alternative cell sizes, or hexagons) to assess tessellation sensitivity. In short, this approach aims at improving comparability and statistical power.

Our approach involves three steps. First, we quantify urban morphology by estimating built form entropy (BFE) from building footprints using a previously proposed measure based on Shannon’s entropy [15]. Second, we measure residential segregation (RS) from the spatial distribution of per-capita income using Moran’s *I* (local spatial autocorrelation). Third, we assess the relationship between BFE and RS by correlating their variations across the study area (Fig 1). Residential segregation measurement is derived from census tracts; we overlay the same grid to ensure comparability and obtain a stable depiction of local income composition. The collection and analysis method complied with the terms and conditions for the source of the data.

### Estimating built-form entropy

Urban morphology was characterised by reducing the built form to two-dimensional arrangements based on building footprints. These figure-ground diagrams, also known as Nolli maps, can be explored to analyse and study the urban form based on built/unbuilt distinctions. Building footprints were extracted from the São Paulo municipality map obtained from GeoSampa and OpenStreetMap services. Considering a trade-off between resolution and data availability [15,16], we chose to partition the study area into a Cartesian grid with squares corresponding to geographic areas of 9 *km*^2^ (see Fig 1). The image of each square was resized to produce areas covered by 10^3^ cells, each representing a space of 3×3 meters, and converted to a monochrome system. Finally, they were mapped into a matrix of size 1000×1000 cells with binary numerical values, which represent the built/unbuilt distinction. In summary, each square is represented by a bidimensional matrix where each element can be a zero, if it stands for an open space cell, or a one, for a built form cell. We focused our analysis on squares mapping areas of the city with high continuity in built form, avoiding large empty areas or rarefied urbanisation patterns generally present in the more peripheral metropolitan areas of the city. Our method for estimating entropy is well-fitted for continuous urban areas. The high continuity and relative homogeneity of built form allow us to use a specific extrapolation technique that will prove useful for estimating the entropy of two-dimensional sequences.

Shannon’s definition of entropy is based on probabilistic concepts [30]. First, it describes properties of the ensemble of sequences produced by a source. In addition, the source that emits the sequences is characterised by a probability distribution which, in turn, is defined by the set of all possible produced sequences. Since these quantities, in general, are not directly accessible, Shannon already proposed some methods to overcome this difficulty [31]. These approaches are based on the idea that the entropy can be numerically estimated by studying only one very long sequence. For a one-dimensional sequence (a string), a possible approach relies on defining the block entropy of order *n* as:


$$\begin{array}{c} H_{n}=-\sum_{k}p_{n}(k)\log_{2}[p_{n}(k)], \end{array}$$ (1)

where blocks are string segments of size *n*, and the sum runs over all the *k* possible *n*-blocks. Equation (1) corresponds to the Shannon entropy of the probability distribution $p_{n}(k)$. The Shannon entropy density of the considered system, which we indicate with *h*, is obtained from the following limit [32]:


$$\begin{array}{c} h=\lim_{n\to \infty }H_{n}/n, \end{array}$$ (2)

which measures the average amount of randomness per symbol that persists after all correlations and constraints are taken into account. More details about this method can be found in [32,33]. This approach can be generalised to our situation, where we analyse sequences of symbols in two dimensions, as reported in [15,34]. The decisive point consists of defining the *n*-blocks for a two-dimensional matrix. The most intuitive idea is to consider a block of size *n* as a square containing *n* cells. The sequences of $H_{n}$ for *n* values that do not correspond to squares are then obtained by considering blocks that interpolate perfect squares. Note that there is no unique natural way to scan a bidimensional matrix. We tested our approach for different reasonable forms of constructing the blocks, and even for randomly constructed blocks. For matrices representing building footprints, the use of different approaches does not significantly influence the estimation of $H_{n}$ [15,16]. In this analysis, we adopt the block definition introduced in [16], considering blocks up to *n* = 20 (see Fig 2). The limit in equation 2 can be empirically obtained by fitting the $H_{n}/n$ points with an appropriate function and then taking its limit for n→∞. We find heuristically that the following ansatz provides an excellent fit [15]:


$$\begin{array}{c} H_{n}/n\approx a+c/n^{b},\;b,c>0. \end{array}$$ (3)

**Fig 2:**
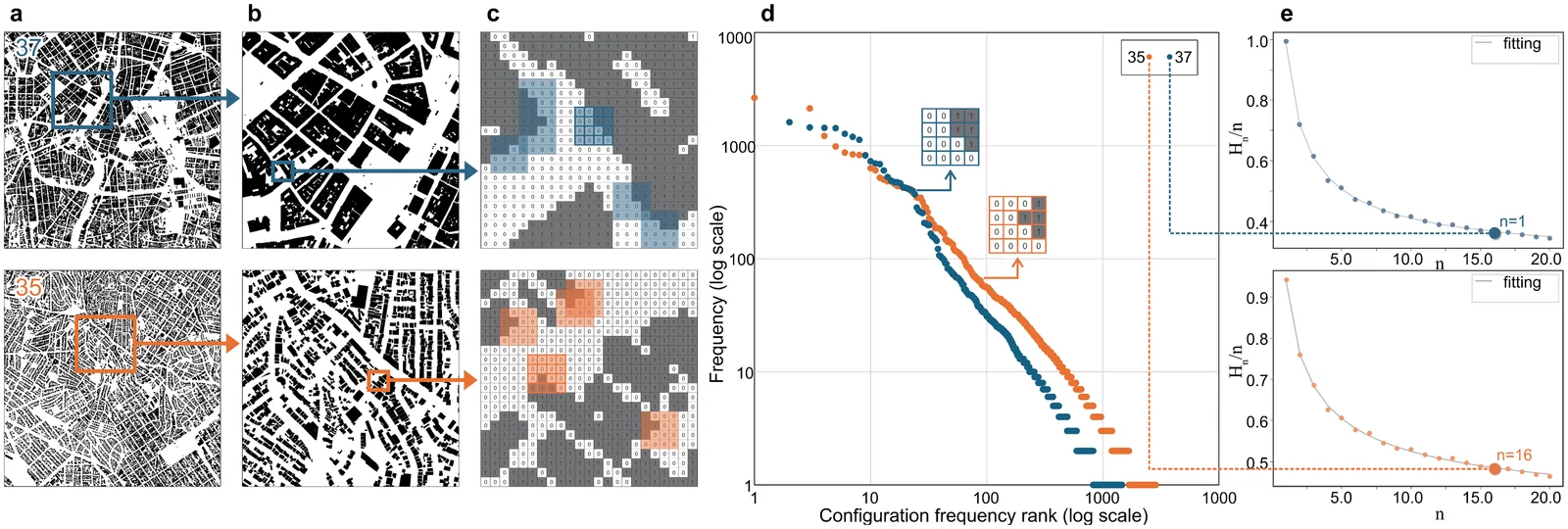
Illustration of the method for estimating built-form entropy (BFE) in cells 37 (top, historic CBD) and 35 (bottom, Pinheiros-Perdizes). (a) The algorithm scans built-form maps in 3×3 km cells and counts the frequencies of local configurations across blocks of different sizes. (b) Zoomed-in views of the building-footprint arrangement. (c) Detection process for a selected configuration with *n* = 16. Insets outlined in blue (cell 37) and orange (cell 35) highlight examples of size-16 blocks. (d) Rank-frequency distribution of built-form configurations for blocks of size 16. (e) Extrapolation of Shannon entropy to infinite block size. The fitted function is: $a+c/n^{b}$ and the estimated parameters are *a* = 0.251, *c* = 0.747, *b* = 0.656 for the cell 37, and *a* = 0.345, *c* = 0.619, *b* = 0.531 for the cell 35. Cell 37 shows more regular arrangements, whereas cell 35 exhibits greater configurational variety.

The fitted value of *a* gives a reasonable extrapolation of the Shannon entropy *h*. Specific considerations on the interpretation and application of this method for characterising the entropy of urban fabric can be found in [15,16,35], generalisations and technical details in [34,36]. By applying this approach, we obtain the value of the Shannon entropy of each square that tessellates the city of São Paulo. Our city contains squares with varying densities of built-form cells. If the density value is far from 50%, there is an important reduction in the entropy value caused just by this asymmetry. This fact is not necessarily connected with randomness and correlations in the built form. For example, the presence of a river or an urban park, which are uniform unbuilt areas, can sensibly reduce the entropy value. As we are interested in characterising the randomness of built form, we introduce a heuristic approach for tackling this problem: we correct the entropy value *h* by adding the term that corresponds to the reduction of entropy due to the asymmetry in the frequency of unoccupied (0) and occupied (1) cells present in the data. Since *H*_1_ is the entropy of the single-cell distribution, the reduction in the value of *h* due to this contribution is equal to $1-H_{1}$. Adding this term to *h* means that, if we do not consider the asymmetry in 0 and 1, the system appears more random by an amount $1-H_{1}$. Such a term will be null in a matrix with perfect equiprobability between 0 and 1. It follows that our corrected entropy is [15]:


$$\begin{array}{c} h_{c}=h+(1-H_{1}). \end{array}$$ (4)

This procedure does not correct all the contributions that an asymmetry in the frequency of 0 and 1 makes to the value of *h* but, at least, it takes away the larger ones. The way *h* differs from *H*_1_ captures in an integrated and involved form how the presence of correlations determines the effective randomness [15].

We applied the Getis-Ord Gi* statistic [37] to the BFE values of the 3×3 km grid cells to identify statistically significant spatial clusters of high entropy (hot spots) and low entropy (cold spots) (Fig 3b). A fixed-distance-band conceptualisation of spatial relationships was adopted, based on Euclidean distance. Because no threshold distance was specified, ArcGIS Pro (Esri) automatically computed and applied a default distance band corresponding to the minimum distance required to ensure that every grid cell had at least one neighbour. For each cell, the Gi* statistic compares the local sum of BFE values within its spatial neighbourhood with the corresponding expectation under spatial randomness and returns a standardised *z*-score. Statistical significance was evaluated at the 90%, 95%, and 99% confidence levels, with a False Discovery Rate (FDR) correction applied to account for multiple local tests. Statistically significant positive *z*-scores identify clusters of high BFE, whereas statistically significant negative *z*-scores identify clusters of low BFE.

**Fig 3:**
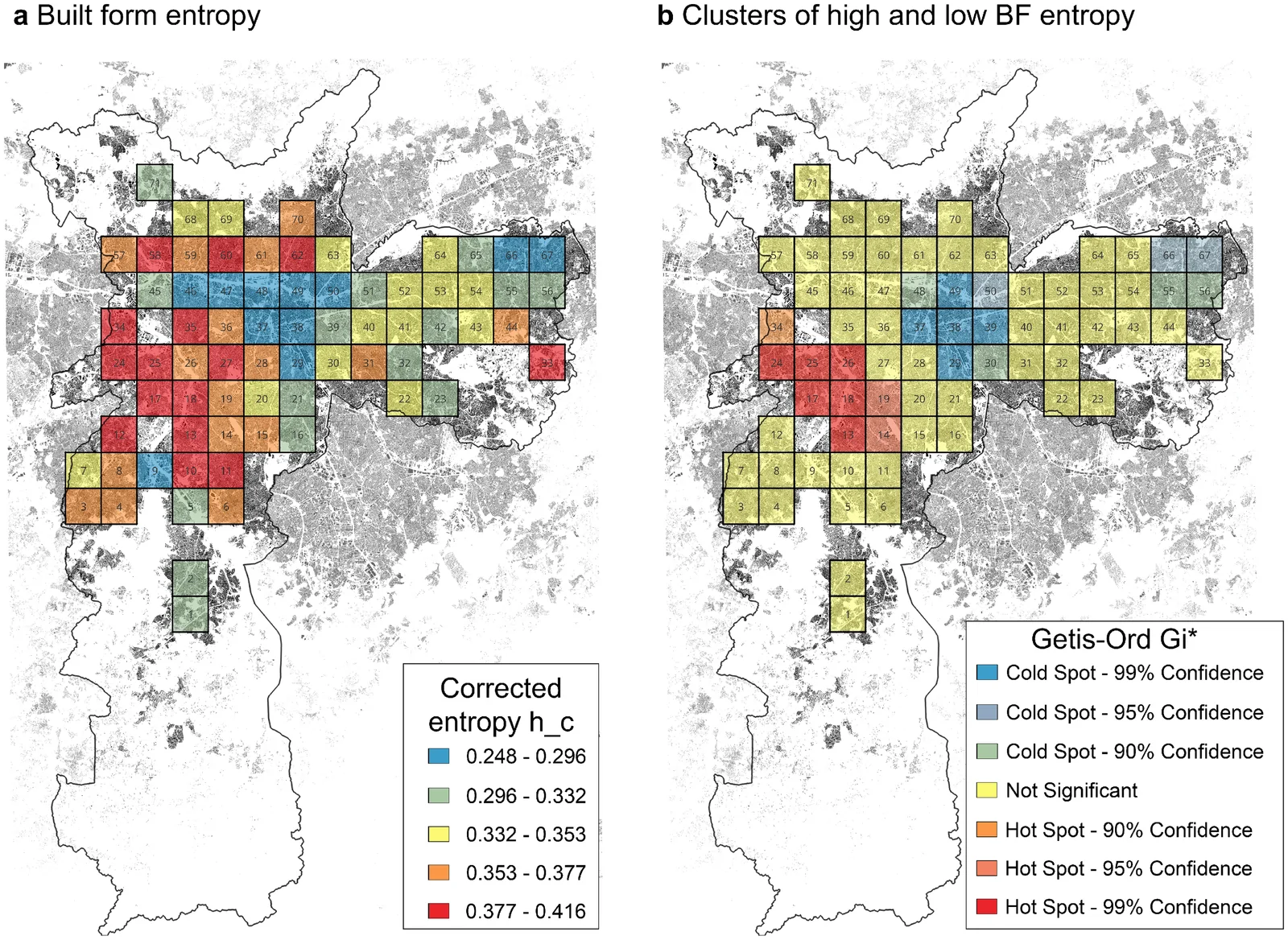
Spatial distribution and clustering of built-form entropy. (a) Building footprint (background) and entropy estimation. The urban fabric exhibits spatial continuity in estimated $h_{c}$. (b) Hot Spot Analysis of BFE with values computed for the 3×3 km grid cells. Red cells indicate statistically significant clusters of high entropy (hot spots), and blue cells indicate clusters of low entropy (cold spots).

### Estimating residential segregation

Social groups were characterised using census-tract population and per-capita income for the municipality of São Paulo from the 2010 Brazilian Census (IBGE) [38]. We used the average per-capita income reported for each tract as our socioeconomic indicator. For descriptive mapping and comparison across the BFE spectrum, grid-cell average per-capita income was classified into five categories using the Jenks natural-breaks procedure, which identifies class boundaries by minimising within-class variance and maximising differences between classes. The resulting intervals were R$ 405–826 (low income), R$ 827–1,436 (lower-middle income), R$ 1,437–2,287 (middle income), R$ 2,288–3,438 (upper-middle income), and R$ 3,439–4,900 (highest income). These labels refer to relative income classes within the analysed São Paulo sample and are used descriptively rather than as official socioeconomic categories.

The Supporting Information illustrates how census tracts relate to a single 3×3 km grid cell in our analysis. We also calculated the Gini index for each grid cell. The Gini coefficient is the most commonly used inequality index. It is a measure of statistical dispersion that, in our case, represents the degree of inequality of income distribution. It is used to characterise how far an income distribution deviates from an equal distribution and ranges from 0 to 1, with 0 indicating perfect equality and 1 representing perfect inequality. We computed Gini coefficients with the R *DescTools* package [39] using the tract-level per-capita income values for all tracts intersecting each grid cell.

Income serves as a proxy for social grouping, so looking at an income map may suggest where segregation is higher or lower. To quantify this intuition, we explored a spatial association index classified by Reardon and O’Sullivan [9] in the ‘spatial evenness’ category. Moran’s *I* is a spatial autocorrelation measure that assesses whether an attribute’s values show clustering, randomness, or dispersion across neighbouring areas. It is well suited to analyse residential segregation, typically characterised by spatial clustering of similar groups. In the context of residential segregation research, Moran’s was first used by Frank [40] to characterise socioeconomic and racial residential patterns in U.S. cities. Using the function *moran.test* of the *Spdep* R package [41,42], we computed Moran’s *I* for each census tract, providing a measure of the local Moran statistic. A global Moran’s coefficient is obtained for each square by averaging all these local values. We first specified the neighbouring polygons and spatial weights. Our primary specification uses a fixed-distance band of 500 m, assigning (equal) weights of 1 to all tracts whose centroids fall within that radius. As a robustness check, we also estimated models with contiguity weights (Queen criterion: polygons sharing at least one vertex). For comparison, we also derived Local Indicators of Spatial Association (LISA) with local Moran and summarised them within cells, which produced patterns consistent with the global statistics.

This coefficient can range between −1 and +1. Positive values indicate that as the income of a given polygon increases, the incomes of its neighbouring polygons tend to increase as well, suggesting the presence of a spatial cluster of high or low incomes. Values close to zero imply a random spatial income distribution, with no apparent clustering. Negative values denote a dispersed spatial pattern, where high-income polygons are surrounded by low-income polygons, and vice-versa, indicating a negative spatial autocorrelation. The values of the Moran’s *I* index and the census tract income estimates can be carried over on the squares of the 3 km × 3 km tessellation using a technique for estimating feature values in overlapping but incongruent polygons (Fig S3c in S1 File). We use the areal weighted interpolation method implemented in R [43]. This method was also used to transfer census tract income estimates to the 9 *km*^2^ squares. Such an approach assumes the variable is uniformly distributed across each spatial unit (3×3 km grid cell). Because cell boundaries are artificial, results are susceptible to MAUP.

## Results

Fig 3 shows corrected-entropy estimates for the square grid of São Paulo. We observe spatial continuity in the values of $h_{c}$, delineating areas with shared morphological characteristics. Corrected entropy exhibits clusters of similar values, with low entropy across the central area (cells 37 and 38) – including parts of districts República, Sé, Bela Vista, Bras, Mooca and Cambuci – and high entropy in districts further south, surrounding cell 13 (e.g., Jaguaré and Santo Amaro – see the complete values per cell for corrected entropy, income, Gini and Moran’s *I* in the Supporting Information).

Fig 4 shows the values of per capita income, Moran’s *I*, and the Gini index for each square. Per capita income provides explicit locational information on social groups, derived by areal-weighting census-tract values onto the square grid. It exhibits a clear centre–periphery pattern: high values cluster in central districts and decline toward the urban fringe, with a broad, relatively uniform low-income area in the east.

**Fig 4:**
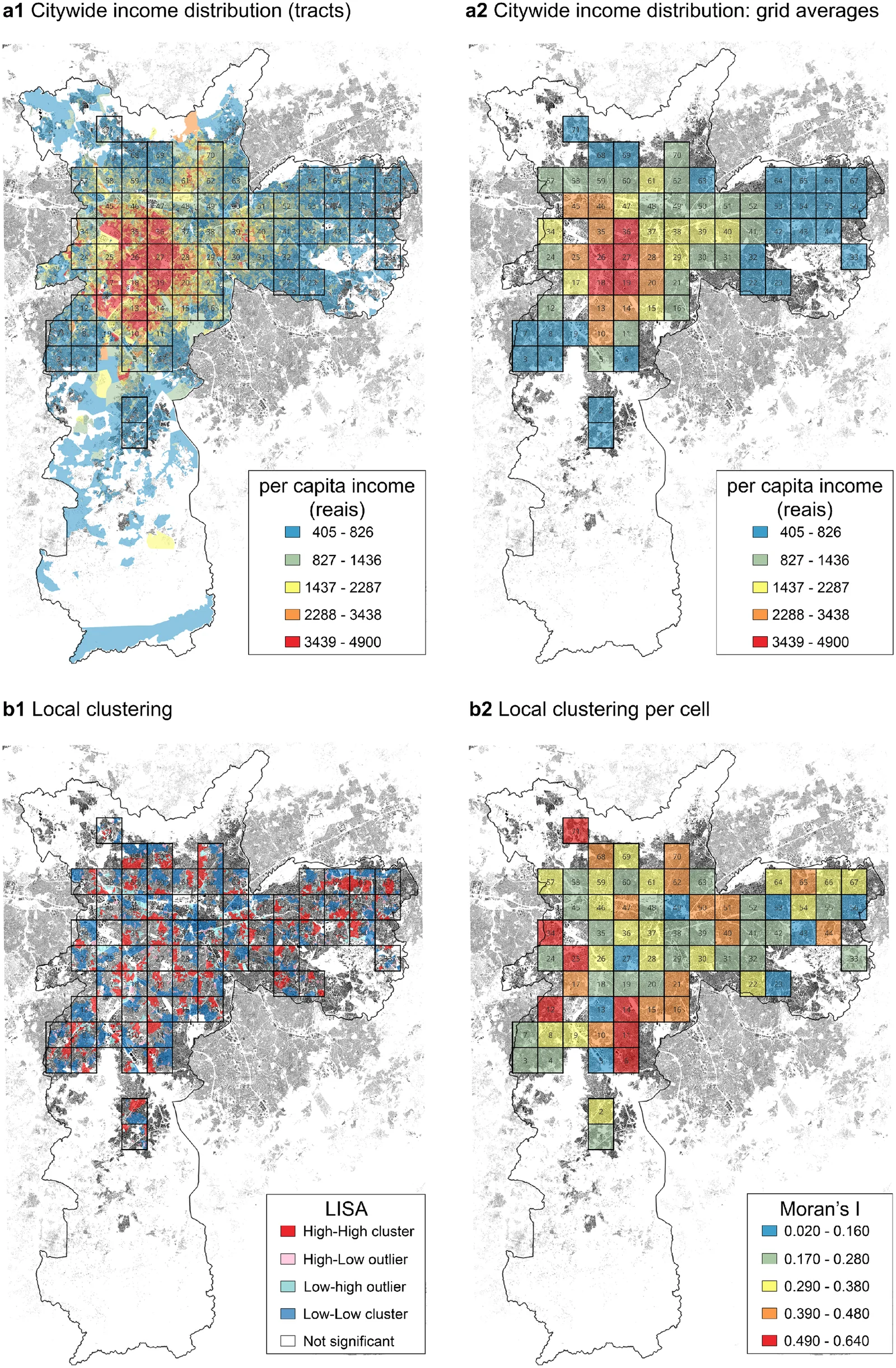
Income and residential segregation in São Paulo. (a1) The citywide distribution of per-capita income by census tract shows a remarkable centre–periphery pattern; (a2) per capita income distribution averaged at the grid cell scale; (b1) Moran’s *I* computed per each 3×3 km grid cell with the Local Indicators of Spatial Association (LISA); (b2) results for Moran’s *I.*

In turn, Gini provides the degree of income inequality between the average per capita income for census tracts within each grid cell. Values are low in the east, where high- and middle-income groups are largely absent, and higher in the area south to (and including) the city centre, where income levels are more mixed. Finally, Moran’s *I* provides an estimation of the spatial autocorrelation using census-tracts values areal-weighted onto the grid cells. In São Paulo, Moran’s *I* displays considerable variation across space and, therefore, requires a more nuanced interpretation.

### Non-linear relationships between built-form entropy and segregation

Having estimated built-form entropy ($h_{c}$) and residential segregation, we ask how they relate. If segregation is intertwined with urban morphology, our entropy measure should capture part of that relationship by quantifying the randomness in building-footprint configurations. We therefore examine whether systematic spatial associations emerge between built-form entropy and residential segregation across São Paulo. At first glance, these variables appear to be only weakly related. Yet, the relationship clearly displays a threshold behaviour. We begin by examining the scatterplot of the corrected BF entropy against income (Fig 5a-b).

**Fig 5:**
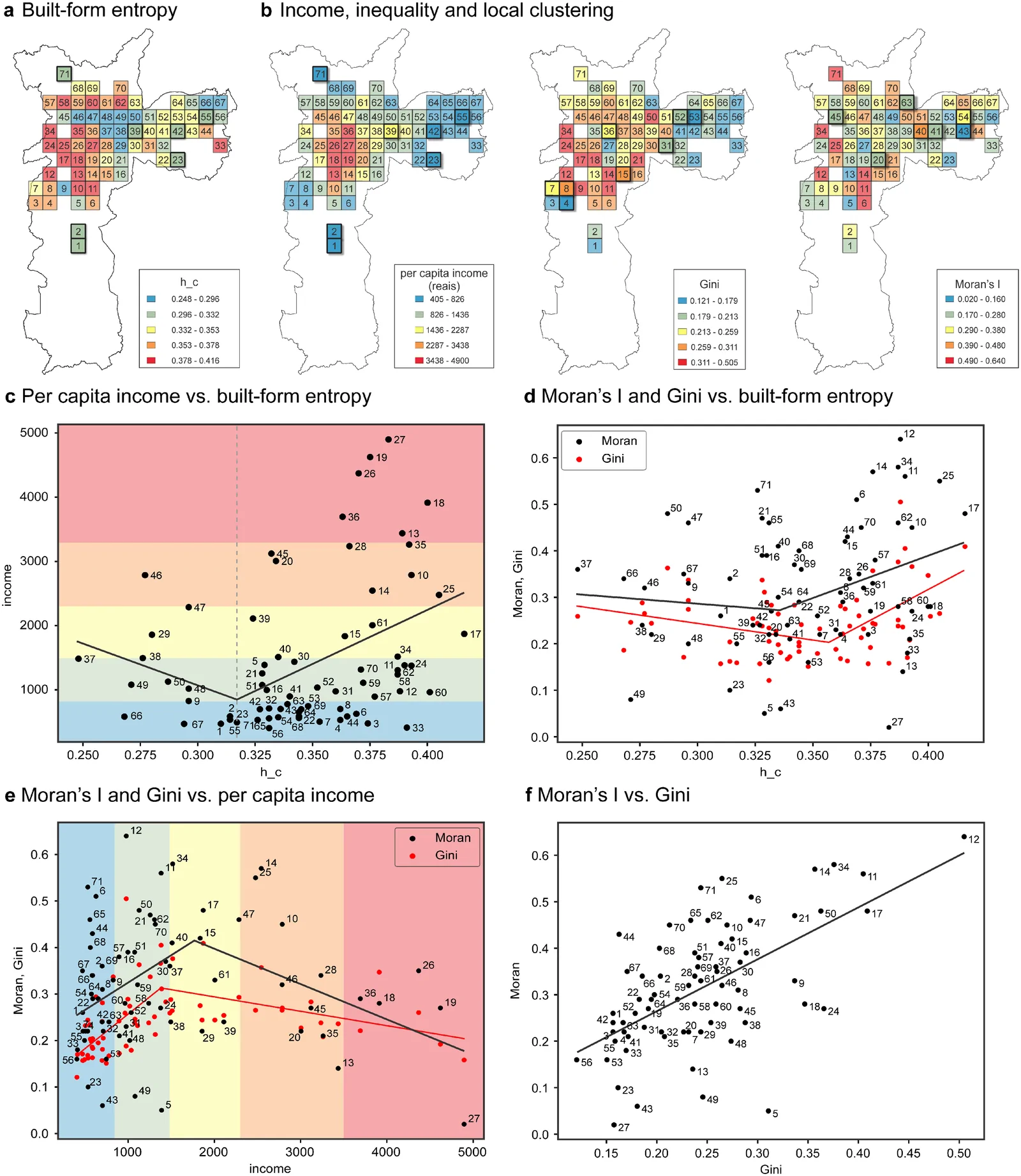
Built-form entropy, income distribution and local residential segregation in São Paulo. Top panels: (a) corrected built-form entropy ($h_{c}$) per grid cell; (b) average per-capita income, income inequality (Gini) and residential segregation (Moran’s *I*) on the same grid. Cells outlined with thicker black lines in (a) and (b) mark areas whose values lie near the estimated threshold. (c) Scatterplot of per-capita income versus $h_{c}$: the solid line shows the piecewise regression; the vertical dashed line identifies the estimated entropy threshold $h_{c}^{T}\sim 0.317$. (d) $h_{c}$ versus Moran’s *I* (black) and Gini (red); solid line, piecewise regression. Cell numbers corresponding to Gini values are reported in the Supporting Information. (e) Income versus Moran’s *I* (black) and Gini (red); solid lines, piecewise regression. (f) Moran’s *I* versus Gini; solid line, best linear fit (*r* = 0.59; *p* < 0.01). The fitting results are detailed in Table 1.

For entropy values below a threshold, the corresponding income is generally moderate or low; by contrast, above that threshold, very high incomes become more likely. We quantify this pattern with a piecewise (segmented) regression [20]. We adopted this model because, despite its simplicity, it is highly effective at capturing and identifying thresholds. Our dataset exhibits high dispersion and our main focus is to detect the threshold separating two distinct regions where points abruptly display a different distribution (e.g., certain value intervals are excluded in one but populated in the other). Specifically, we fit a simple broken-stick model – two linear segments joined at an unknown breakpoint – where the estimated breakpoint defines the $h_{c}$ threshold. The best-fitting broken-stick model yields a first segment with a negative slope and a second with a positive one. At low $h_{c}$, income is moderate and declines as $h_{c}$ increases. Once the estimated threshold $h_{c}^{T}\sim 0.317$ is reached, the relationship reverses: beyond this threshold, higher $h_{c}$ produce an increase in income values. In short, the association is negative below the threshold and positive above it, with income attaining its minimum around $h_{c}^{T}$. To determine whether the relationship contains a breakpoint where the slope changes or it is strictly linear, we compared a standard linear model against the broken-stick model using the Akaike information criterion (AIC). The model with the lowest Akaike information is the best supported model. The broken-stick model yielded the lower AIC value, confirming the importance of introducing the breakpoint. Details on the statistical analysis are presented in Table 1.

**Table 1:** Details of the fitting results. β is the breakpoint of the piecewise regression, $\alpha_{1}$ and $\alpha_{2}$ are the slopes of the two lines, *AIC*_0_ is the *AIC* for a linear model and *AIC*_1_ for the piecewise regression. Overall, the estimated slopes are statistically significant, save for two cases where the sub-sample sizes to the left of the breakpoint, in addition to exhibiting the natural high variance, are quite small. Specifically, for Moran vs. $h_{c}$, a piecewise regression with a zero-slope constant term to the left of the breakpoint provides better statistical support. Regardless, this does not alter the qualitative discussion of our results.

variables	β	$\alpha_{1}$; *p*-*value*	$\alpha_{2}$; *p*-*value*	*AIC*_0_	*AIC*_1_
income vs $h_{c}$	0.317±0.022	$(-1.31\pm 3.54)\cdot 10^{4}$; 0.42	$(1.68\pm 0.56)\cdot 10^{5}$; 0.004	971	966
Gini vs $h_{c}$	0.357±0.009	−0.71±0.39; 0.07	2.62±0.83; 0.003	−357	−371
Moran vs $h_{c}$	0.336±0.025	−0.39±0.97; 0.68	1.81±1.03; 0.08	−274	−277
Gini vs income	1383±154	$(1.48\pm 0.33)\cdot 10^{-4}$; $3\cdot 10^{-5}$	$(-3.08\pm 1.09)\cdot 10^{-5}$; 0.006	−357	−387
Moran vs income	1774±354	$(1.17\pm 0.52)\cdot 10^{-4}$; 0.03	$(-7.60\pm 3.09)\cdot 10^{-5}$; 0.02	−271	−282

An analogous threshold behaviour can be observed when plotting the Gini or Moran’s *I* as a function of entropy (Fig 5e). The estimated breakpoint again lies near the median $h_{c}$. Below the threshold, entropy is likely to cause a marginal decrease in both indices, which reach their lowest values at the threshold. Above it, both indices increase. The parallel behaviour is consistent with the strong correlation between Gini and Moran’s *I* (see Fig 5f). The Gini index clearly increases with Moran’s *I*, indicating that higher income inequality is accompanied by stronger spatial segregation. Finally, we analysed how Gini and Moran’s *I* vary with income. Both exhibit a threshold behaviour: the fitted piecewise regressions have a positive slope at lower incomes and a negative slope beyond the estimated breakpoint. Most of the highest values of both indices occur below the breakpoint, while at higher incomes they decline (Fig 5e). In other words, above the income threshold, higher incomes coincide with the absence of high Gini and Moran’s *I* values, indicating greater income equality across the tracts composing each grid cell and weaker local clustering relative to neighbouring cells.

Together, these patterns indicate a systematic, non-linear spatial alignment between built-form entropy, income, and residential clustering. Table 1 provides the detailed outputs of these latter statistical analyses, including the AIC comparison between the broken-stick and the linear models.

## Discussion

In line with accounts of residential segregation as a scale-dependent phenomenon [21–23] and with location theory since Alonso [24] (e.g., [25,26]), São Paulo shows a pronounced centre–periphery pattern of income-based residential distribution shaped by citywide locational forces, alongside more localised manifestations of segregation expressed as clustering of different income groups within grid cells. These two expressions are analytically distinct: large-scale income concentrations can dominate entire areas without necessarily generating high levels of within-cell spatial clustering, whereas local clustering may emerge even within broader income gradients. Against this backdrop, we hypothesised that variation in built-form entropy is associated with the morphological conditions under which income groups cluster locally. Specifically, in areas where different income groups coexist, higher built-form entropy tends to be associated with stronger local clustering (H1); income groups are unevenly distributed across the entropy spectrum, with some groups concentrating in specific entropy regimes (H2); the associations of built-form entropy, income and residential segregation are non-linear (H3). To clarify how the hypotheses map onto the empirical patterns, we identify a set of recurrent regimes in the wider continuum of cases associated with the threshold-based relationship between built-form entropy, income inequality, and local clustering.

- First, high built-form entropy, high Gini, and high Moran’s *I* correspond most directly to H1: morphologically complex areas in which multiple income groups coexist and cluster locally. We find that very high Moran’s *I* values occur only in high built-form entropy cells, indicating the co-occurrence of distinct income clusters associated with differentiated morphologies at the local scale. In our data, this regime includes cells 11, 12, 14, 17, and 34 (Fig 6a). Cases where high BFE meets high residential clustering include cells 6, 10, 25 and 62, at medium Gini values.
- Second, a regime of high built-form entropy combined with low Gini and low or low-to-medium Moran’s *I* corresponds most directly to H2. This configuration captures situations where a particular income group concentrates within a specific entropy regime, in an area with relative internal income equality and limited local clustering. It includes lower-income cells 3 and 33, and higher-income cells 19 and 35, and 27 as a limiting case characterised by the dominance of the highest-income group and minimal internal clustering (Fig 6b).
- Third, cells with intermediate built-form entropy, low Gini, and low Moran’s *I* correspond to a near-threshold regime in which both inequality and clustering remain comparatively low; this regime includes lower-income cells 23, 43, 53, 55, and 56 in eastern São Paulo (Fig 6c).

**Fig 6:**
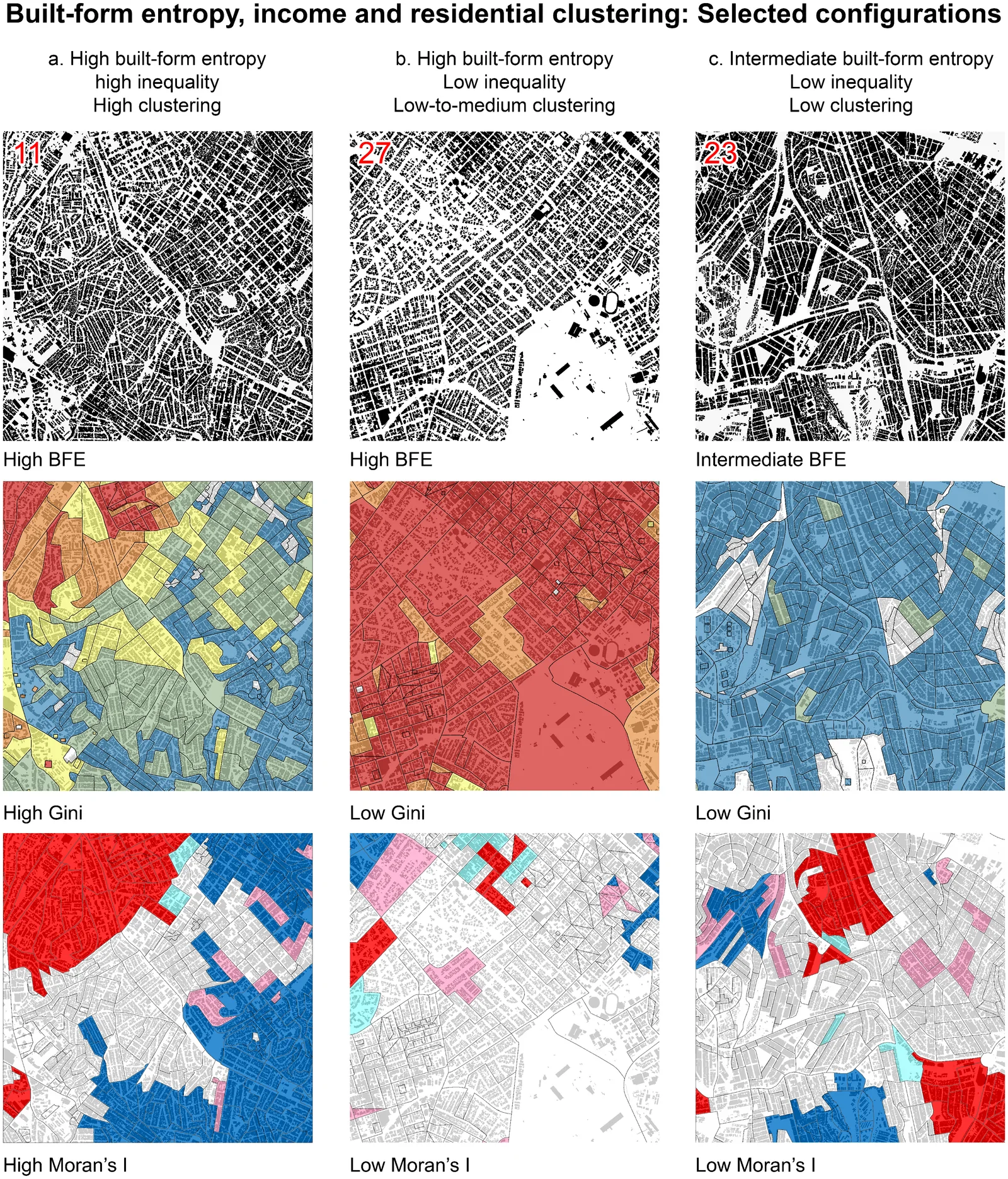
Selected configurations of built-form entropy, income distribution, and residential clustering. Each column shows a 3×3 km cell. (a) Cell 11 illustrates high built-form entropy, high Gini, and strong local clustering, where income clusters co-occur with contrasting morphologies. (b) Cell 27 represents a limiting case of high built-form entropy with a nearly homogeneous high-income group and low internal clustering. (c) Cell 23 shows an intermediate-entropy, lower-income configuration with low inequality and low clustering.

A contrasting threshold-related configuration is formed by cells with intermediate built-form entropy, and medium-to-high Moran’s *I* and Gini – namely cells 16, 21, 30, 40, 51, 65, 68, and 71 – showing that areas near the threshold may also coincide with local clustering and uneven income composition (Fig 6d). A configuration with low built-form entropy, high Gini and high Moran’s *I* includes only two cells, 47 and 50, showing that ordered built form may also coincide with internal inequality and strong local clustering. The regimes observed above do not exhaust all possibilities or empirical cases, but represent specific configurations that add nuance and complexity to the broader field defined by the non-linear relationship between built-form entropy and the inequality and clustering measures. This trend is also evident at the low-entropy end of this non-linear pattern: configurations with low built-form entropy, medium Moran’s *I*, and medium Gini show that ordered built form may also coincide with some internal inequality and local clustering; this regime includes cells 9, 37, 38, 46, 66, and 67, in line with the interpretation that built-form entropy is associated with a structured range of outcomes rather than a single segregation pattern.

### Interpreting built-form entropy and residential segregation patterns

Per-capita income in São Paulo follows a pronounced centre–periphery gradient radiating from the area southeast of the historic Central Business District (CBD), reflecting a highly segregated urban structure in which income strongly conditions residential location. Within this broader pattern, our results indicate systematic associations between built-form entropy and the spatial distribution of income groups (Fig 5c). Income groups do not locate randomly across the built-form entropy (BFE) spectrum; rather, they tend to concentrate in distinct entropy bands:

- Low income (R$ 405–826; blue; 26 cells): although present across the full BFE range, most cells lie above the threshold $h_{c}^{T}\sim 0.317$. Only 1 of 26 (cell 33) occurs above $h_{c}\sim 0.374$, indicating that low-income locations concentrate mainly in the intermediate-to-high entropy range between 0.317 and 0.374
- Lower-middle income (R$ 827–1,436; green; 20 cells): 9 of 20 cells lie above $h_{c}\sim 0.374$, while only 4 of 20 fall below the BFE threshold $h_{c}^{T}\sim 0.317$, indicating a clear a tilt toward higher-entropy contexts.
- Middle income (R$ 1,437–2,287; yellow; 10 cells): these cells are distributed broadly across the BFE range, with no sharp concentration, consistent with a transitional position in both income and morphology.
- Upper-middle income (R$ 2,288–3,438; orange; 9 cells; avg. per-capita income): although present at several BFE values, this group concentrates in high-entropy areas: only 3 of 9 upper-income cells fall below $h_{c}\sim 0.363$, increasing in count as $h_{c}$ rises.
- Highest income (R$ 1,437–2,287; red; 6 cells): all cells in this category lie above $h_{c}\sim 0.363$ (see Fig 5c).

To ground the broader patterns in concrete urban contexts, Fig 7 contrasts selected cells from the high- and low-entropy clusters. The high-entropy cases include affluent areas such as Morumbi (cell 17), Pinheiros–Perdizes (cell 35), and Jardim Paulista (cell 27), where intricate morphologies coincide either with strong local clustering or with socially homogeneous high-income contexts, as well as mixed or lower-income areas such as Jaguaré (cell 34), where morphological irregularity is also associated with clustered social differentiation. By contrast, the low-entropy cases include the historic CBD in República-Sé (cell 37) and eastern peripheral areas such as São Miguel and Jardim Helena (cells 66–67), where regular block systems and greater morphological continuity coincide with weaker clustering or more moderate internal differentiation. These examples provide empirical illustration for the broader associations described above. More detailed historical and contextual notes on these and other areas are included in the Supporting Information.

**Fig 7:**
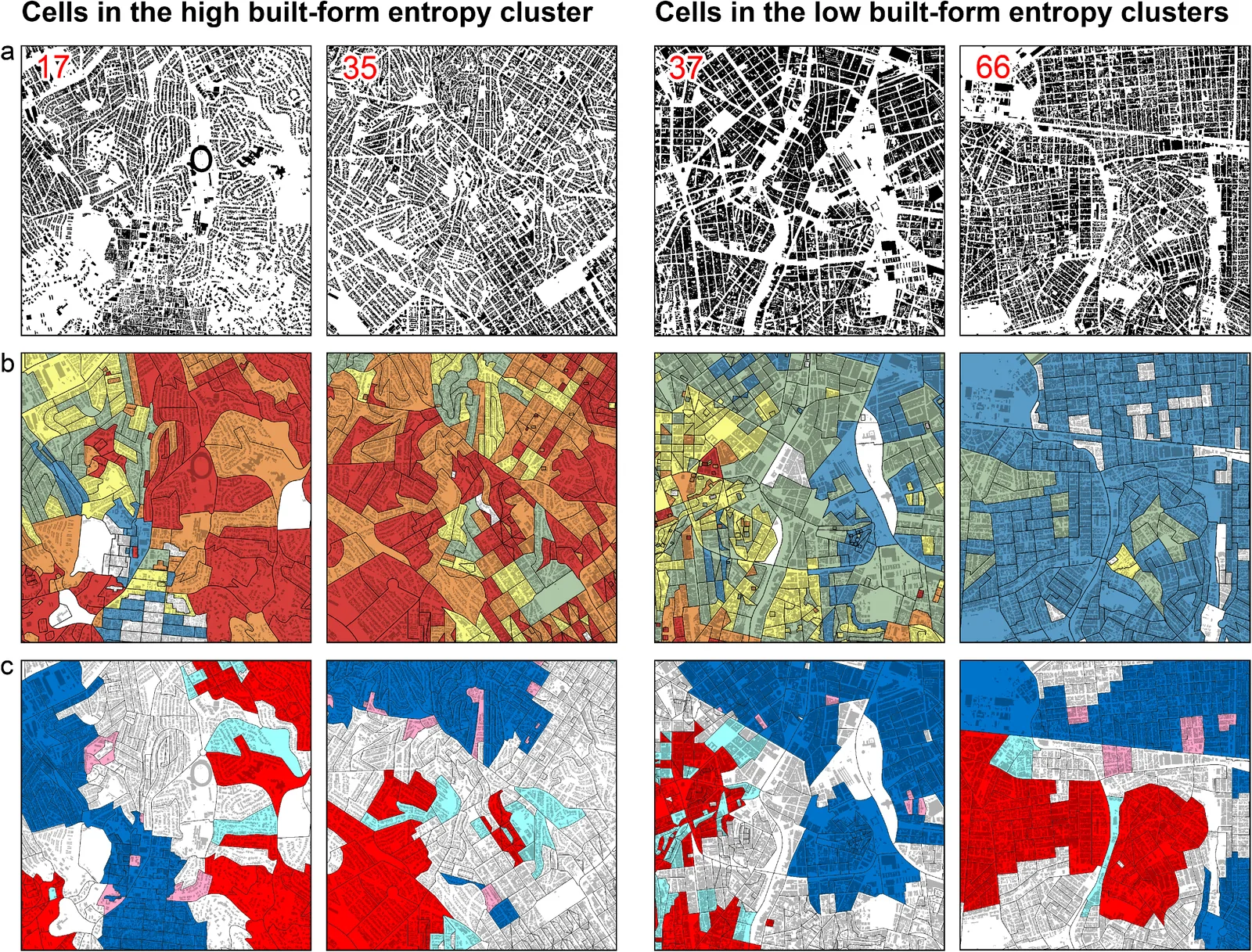
Selected 3 × 3 km cells from the high-entropy cluster (cells 17 and 35) and the low-entropy clusters (37 and 66). Rows show: (a) built form distributions; (b) tract-level average per-capita income, and (c) local residential clustering of income groups (Moran’s *I*). Cell 17 (Vila Sonia – Morumbi) ranks first citywide in BFE, while Morumbi has the highest average per capita income. Cell 35 (Pinheiros–Perdizes) ranks eighth in BFE and eighth in income. Cell 37 (República–Sé) includes the historic CBD; despite a pronounced angular variation in its street network, its compact perimeter blocks with aligned façades yield the lowest BFE in the city. Cell 66 (São Miguel, Jardim Helena, north of Vila Curuçá) sits on the far eastern periphery – a low-income area with some regular block systems.

At the cell scale, higher BFE generally coincides with stronger local clustering, while high-income groups are concentrated above the estimated entropy threshold (Fig 5d). These areas display distinct but equally entropic morphologies: low-rise, detached housing embedded in winding street layouts, and vertically intensified fabrics of high-rise buildings. Both forms are typically shaped by planning regimes that privilege parcel-level design over coordinated construction, allowing substantial variation in building footprint and encouraging piecemeal development. In most newly urbanised areas, the requirements for continuity with surrounding street networks are weak, which further contribute to built-form fragmentation. Such configurations tend to reduce legibility, permeability, and external accessibility, operating as “soft barriers” that can moderate exposure between social groups (cf. [18,27]). In contrast, lower-income groups span a range of entropy values but show a clear tendency toward higher entropy, consistent with the morphological variability, including informal urbanisation and self-produced settlements. Uneven planning histories and incremental urbanisation processes may also funnel low- and lower-middle-income groups into mid-to-high entropy fabrics shaped by partial formalisation, while higher-income groups concentrate in high-entropy environments that offer both distinction and insulation.

A notable feature of the threshold-based relationship is the asymmetry across the entropy spectrum. At the high-entropy end, distinct social logics converge on similarly high BFE values: elite enclaving and lower-income settlements each generate morphological irregularity through different urban processes: the former through designed detached housing, high-rise buildings, and winding street layouts, the latter through self-built, incremental, and weakly regulated development. This helps explain why the high-entropy end accommodates both H1-type outcomes, in which multiple income groups coexist and cluster locally, and H2-type outcomes, in which a single income group concentrates in a specific entropy regime and dominates a morphologically complex area.

At the same time, the pattern is not confined to the high-entropy side. At the low-entropy end, both income and residential clustering also rise, albeit less steeply, indicating that relatively affluent areas and moderately clustered configurations may also occur in more ordered morphologies. The asymmetry is twofold: high entropy concentrates a broader range of socio-spatial processes and more intense clustering outcomes, whereas low entropy is compatible with selective concentration and moderate segregation, though through a narrower or weaker set of processes. The same entropy value may therefore index different social arrangements, while similar social outcomes may also arise at opposite ends of the spectrum through different morphological and institutional pathways.

One plausible interpretation for the low-entropy side of this asymmetry is the role of planning in producing ordered environments that are attractive to certain higher-income households, alongside zoning and building rules that contribute to higher land values and selective occupation. Unlike the high-entropy end, where morphological irregularity may operate as a soft barrier, ordered built form may be associated with segregation through regulatory and economic filtering rather than through spatial intricacy. These area-specific conditions call for finer-grained contextual research than citywide associational analysis can provide.

The built-form entropy and inequality relationship is also non-linear: Gini reaches its minimum near the entropy threshold $h_{c}^{T}\sim 0.358$, declining weakly below it and increasing above it, indicating a tipping region in the BFE–Gini association (Fig 5d; S2 Fig in S1 File). These results suggest that cells containing multiple income groups often coincide with more irregular built-form configurations at the local scale.

Beyond built-form entropy, our data show that per-capita income, the Gini index, and Moran’s *I* are related in two ways (Fig 5e). First, both Gini and Moran’s *I* peak at middle income levels: at the extremes, high-income areas tend to exclude low-income residents (and vice versa), which reduces within-cell heterogeneity and local autocorrelation, resulting in lower values for both indices. Second, identical Gini values can arise from very different spatialisations – dispersed, banded, or tightly clustered – so income composition (Gini) and spatial arrangement (Moran’s *I*) need not coincide in principle. However, in the study area the two indices show a moderate positive association (Pearson’s *r* = 0.59; *p* < 0.01; Fig 5f), indicating that income unevenness and local residential clustering tend to co-occur. This relative alignment is consistent with well-established segregation dynamics in which households’ locational preferences – often modest at the individual level – aggregate into spatial clustering of similar groups, as classically illustrated by Schelling’s model of residential segregation [28] (see Supporting Information). Under such conditions, uneven income composition within cells is likely to be spatially expressed through clustering in neighbouring tracts, reinforcing the correspondence between Gini and Moran’s *I*. Although this relationship is not logically necessary – since similar levels of inequality can be achieved under different spatial arrangements – it is nonetheless a frequent outcome in settings shaped by income unevenness, land-value gradients, and segregation processes.

## Conclusions

This study set out to clarify how the morphology of the built environment relates to residential segregation. By placing built-form entropy (BFE) and residential segregation (RS) metrics on a common spatial footing, we find that urban form is systematically associated with income-based residential distribution and local clustering, and that these associations are non-linear and threshold-sensitive. The conclusions below synthesise these findings in relation to the complementary hypotheses introduced earlier.

(1) Examining the relationship between built-form entropy and residential segregation, we find that highly entropic areas more often exhibit stronger local income clustering – that is, areas in which income groups co-occur but are internally clustered (Fig 5d), in line with our first hypothesis (H1).
(2) Income groups are not evenly distributed across the built-form entropy spectrum. Instead, they occupy distinct entropy ranges, indicating that the association between built-form entropy and residential segregation varies across income levels (Fig 5c). The clearest pattern is that high-income groups are concentrated in high-entropy areas, overwhelmingly located well above the estimated entropy threshold at $h_{c}^{T}\sim 0.317$. In turn, lower-income groups span a broader range of entropy values, but tend to concentrate above the threshold in moderately to highly entropic areas, a pattern even more marked among lower-middle-income groups. Under certain conditions, these distributions may also produce socially homogeneous areas with low internal clustering. These results are consistent with our second hypothesis (H2).
(3) The relationship between built-form entropy and residential segregation, captured by Moran’s *I*, is distinctly non-linear: local clustering reaches its minimum at intermediate entropy values and increases toward both lower and higher-entropy contexts, with a steeper increase at high entropy. The most segregated areas tend to coincide with higher built-form entropy, typically associated with morphologically intricate fabrics composed of fragmented block systems and fine-grained mixtures of building volumes. The relationships between BFE and per-capita income (Fig 4c) and income inequality (Figs 4d, S2 Fig in S1 File) show similar non-linear behaviour (H3).

The asymmetry of the piecewise regression, with a steeper slope at the high-entropy end than at the low-entropy end, together with the different dispersion of data points at these extremes, suggests that distinct socio-spatial processes converge on related entropy signatures. Elite enclaving may associate high built-form entropy with high-income clustering, while weakly regulated urbanisation may associate similarly high entropy with lower-income clustering. By contrast, the low-entropy end appears to reflect a narrower set of processes plausibly linked to planning, amenity attraction, and regulatory filtering. The high-entropy flank presents a larger dispersion of data points, which may reflect the broader range of socio-spatial processes converging there, and a steeper slope, suggesting that some of these forces may be more intense. This interpretation helps explain why soft barriers of reduced permeability and intervisibility may be more strongly expressed at the high-entropy end than attractions and planning constraints associated with more ordered built form.

These effects align with our general proposition that morphological entropy is associated with spatial conditions under which social segregation is expressed as residential clustering. Under a null expectation of no systematic spatial alignment, the placement of income groups across the built-form entropy spectrum would show no systematic structure, and our metrics would not vary coherently with entropy. Built form appears to be associated with patterns of social distinction between areas and social homogeneity within them, while potentially providing reduced visibility, accessibility, and intergroup exposure (cf. [19,29] on street networks). In this sense, our findings are consistent with an interpretation of urban morphology not as a passive backdrop, but as an active component in the social organisation of residential space – both signal and affordance of segregation. In particular, the co-occurrence of high entropy, high average income, and high Moran’s *I* suggests that built form may help structure residential distributions at this scale rather than merely reflect them. Together, citywide income gradients and localised morphological variation signal scale-dependent processes: micro-level residential clustering coexists with macro-level income-based residential distribution, adding complexity to the interaction of segregation processes and urban form.

The consistent turning point around $h_{c}^{T}\sim 0.317$ highlights threshold sensitivity and calls for alternative analyses of urban form. Future work should examine whether this threshold is stable across cities with different built-form structures, income distributions, and planning histories, or whether it is a property specific to São Paulo’s particular morphological and socio-economic configuration. The analysis nonetheless remains subject to scale and data sensitivities. Results may vary in relation to MAUP. We work with a 3×3 km lattice. Cell sizes were tested and the applied cell size shows the required robustness in both entropy measurement and local sampling of census tracts. Areal-weight income transfers from census tracts, so the position of the tessellation can slightly shift Gini, Moran’s *I* and their correlations with $h_{c}$. The tessellation origin effect – moving the grid a few hundred metres – can change which tracts fall into which cells and thus alter within-cell heterogeneity and local clustering. Notwithstanding, effects of shifts in tessellation position are likely to be balanced out within the overall pattern of income and BF distribution.

Finally, the study is associational: piecewise fits and correlations indicate systematic co-location, not causation. However, the consistent co-location of high built-form entropy and strong residential segregation clearly go beyond what a random allocation would produce. Two non-exclusive mechanisms are plausible for explaining these effects: selection, in which income groups preferentially locate in high-entropy fabrics to limit visibility, access, and contact; and production, in which groups (and institutions) shape morphology to achieve the same effects. Identifying the dominant pathway lies beyond this study, but the overlap suggests that morphology may be actively involved in segregation dynamics. Future work should replicate the study across multiple cities and time slices in comparative and longitudinal tests (e.g., pre/post zoning changes) to assess transferability and contextual dependence. Even so, the observed patterns point to potential practical levers in planning agendas: changes in urban block arrangement and permeability, parcel structure, and building configuration may modify the spatial conditions associated with segregation, even where citywide income gradients persist. An applied research path involves examining the mediating role of planning interventions such as zoning reforms and redevelopment policies, addressing questions like: can morphology guided by planning lead to changes in residential segregation – say, may the adoption of more systemic, form-based codes prioritising spatial criteria such as continuity in urban block systems and building placement decrease morphological entropy? Such efforts could also clarify why similar income mixes produce different segregation outcomes under different physical fabrics, and whether targeted morphological interventions can meaningfully reconfigure clustering, exposure, and segregation dynamics.

Beyond the empirical findings, this study aims to contribute in four main ways. First, it brings the physical fabric into residential segregation analysis as a structural property examined alongside inequality and segregation indices. Second, it identifies the form of association between built form and residential segregation, including thresholds that indicate turning points in the relationship. Third, it addresses scale dependence, showing citywide income gradients and local clustering under different morphological entropy regimes. Finally, it offers a replicable workflow, from building footprints to built-form entropy, segregation indices, and association tests, that can be applied to other cities and contexts.

## Supporting information

S1 File Supporting Information (S1.pdf file) includes an extended literature review, grid-level measurement of built-form entropy, income and residential segregation indicators, full piecewise regression results for the entropy-Gini relationship in São Paulo, and contextual notes on high and low entropy clusters. (PDF)

## References

[1] Burgess EW. Residential segregation in American cities. Ann Am Acad Political Soc Sci. 1928;140:105–15. doi: 10.1177/000271622814000115

[2] Wong DW. Formulating a General Spatial Segregation Measure. Prof Geogr. 2005;57:285–94.

[3] Netto V, Krenz K, Fiszon M, Peres O, Rosalino D. Decoding segregation: Navigating a century of segregation research across disciplines and introducing a bottom-up ontology. arXiv preprint arXiv:2410.08374. 2024.

[4] Booth C. Poverty maps of London. 1889.

[5] Balch EG. Hull house maps and papers. 1895.

[6] Duncan OD, Duncan B. A Methodological Analysis of Segregation Indexes. Am Sociol Rev. 1955;20(2):210. doi: 10.2307/2088328

[7] James DR, Taeuber KE. Measures of Segregation. Sociol Methodol. 1985;15:1–32. doi: 10.2307/270845

[8] Theil H, Finizza AJ. A note on the measurement of racial integration of schools by means of informational concepts†. J Math Sociol. 1971;1(2):187–93. doi: 10.1080/0022250x.1971.9989795

[9] Reardon SF, O’Sullivan D. 3. Measures of Spatial Segregation. Sociol Methodol. 2004;34(1):121–62. doi: 10.1111/j.0081-1750.2004.00150.x

[10] Morrill R. On the measure of geographic segregation. In: Geography research forum, vol. 11. 1991. p. 25–36.

[11] Wong DWS. Spatial indices of segregation. Urban Studies. 1993;30:559–72. doi: 10.1080/00420989320080551

[12] Vaughan L. The relationship between physical segregation and social marginalisation in the urban environment. World Architecture. 2005;185:88–96.

[13] Vaughan L. The spatial syntax of urban segregation. Progress Planning. 2007;67(3):205–94. doi: 10.1016/j.progress.2007.03.001

[14] Saboya RT de, Peres OM. CHASM: A configurational measure of socio-spatial residential segregation. Environ Plann B. 2024;52(6):1464–81. doi: 10.1177/23998083241287701

[15] Brigatti E, Netto VM, de Sousa Filho FNM, Cacholas C. Entropy and hierarchical clustering: Characterizing the morphology of the urban fabric in different spatial cultures. Chaos. 2021;31(11):113138. doi: 10.1063/5.0069258 34881605

[16] Netto VM, Brigatti E, Cacholas C. From urban form to information: Cellular configurations in different spatial cultures. Environ Plann B. 2022;50(1):146–61. doi: 10.1177/23998083221107382

[17] Hillier B, Penn A, Hanson J, Grajewski T, Xu J. Natural movement: or, configuration and attraction in urban pedestrian movement. Environ Plann B. 1993;20(1):29–66. doi: 10.1068/b200029

[18] Roberto E, Korver-Glenn E. The Spatial Structure and Local Experience of Residential Segregation. Spat Demogr. 2021;9(3):277–307. doi: 10.1007/s40980-021-00086-7

[19] Hillier B, Hanson J. The social logic of space. Cambridge University Press; 1984.

[20] Toms JD, Lesperance ML. Piecewise regression: a tool for identifying ecological thresholds. Ecology. 2003;84(8):2034–41. doi: 10.1890/02-0472

[21] Johnston R, Jones K, Manley D, Owen D. Macro‐scale stability with micro‐scale diversity: modelling changing ethnic minority residential segregation – London 2001–2011. Trans Inst British Geog. 2016;41(4):389–402. doi: 10.1111/tran.12142

[22] Fowler CS. Segregation as a multi-scalar phenomenon and its implications for neighborhood-scale research: the case of South Seattle 1990-2010. Urban Geogr. 2016;37(1):1–25. doi: 10.1080/02723638.2015.1043775 27041785 PMC4811614

[23] Catney G. The complex geographies of ethnic residential segregation: Using spatial and local measures to explore scale‐dependency and spatial relationships. Trans Inst British Geog. 2017;43(1):137–52. doi: 10.1111/tran.12209

[24] Alonso W. Location and land use: Toward a general theory of land rent. Harvard University Press; 1964.

[25] Brueckner JK, Thisse J-F, Zenou Y. Why is central paris rich and downtown detroit poor?: An amenity-based theory. Eur Econ Rev. 1999;43:91–107.

[26] Guerrieri V, Hartley D, Hurst E. Endogenous gentrification and housing price dynamics. J Public Econ. 2013;100:45–60. doi: 10.1016/j.jpubeco.2013.02.001

[27] Knaap E, Rey S. Segregated by design? Street network topological structure and the measurement of urban segregation. Environ Plan B: Urban Anal City Sci. 2023;51(7):1408–29. doi: 10.1177/23998083231197956

[28] Schelling TC. Dynamic models of segregation†. The J Math Sociol. 1971;1(2):143–86. doi: 10.1080/0022250x.1971.9989794

[29] Roberto E. The Spatial Proximity and Connectivity Method for Measuring and Analyzing Residential Segregation. Sociol Methodol. 2018;48(1):182–224. doi: 10.1177/0081175018796871

[30] Shannon CE. A Mathematical Theory of Communication. Bell Syst Techn J. 1948;27(4):623–56. doi: 10.1002/j.1538-7305.1948.tb00917.x

[31] Shannon CE. Prediction and Entropy of Printed English. Bell Syst Techn J. 1951;30(1):50–64. doi: 10.1002/j.1538-7305.1951.tb01366.x

[32] Schurmann T, Grassberger P. Entropy estimation of symbol sequences. Chaos. 1996;6(3):414–27. doi: 10.1063/1.166191 12780271

[33] Cover TM, Thomas JA. Elements of information theory. John Wiley & Sons, Inc.; 1991.

[34] Brigatti E, de Sousa Filho FNM. Comment on “Universal and Accessible Entropy Estimation Using a Compression Algorithm”. Phys Rev Lett. 2022;129(2):029801. doi: 10.1103/PhysRevLett.129.029801 35867442

[35] Netto VM, Brigatti E, Meirelles J, Ribeiro FL, Pace B, Cacholas C, et al. Cities, from Information to Interaction. Entropy (Basel). 2018;20(11):834. doi: 10.3390/e20110834 33266557 PMC7512396

[36] de Sousa Filho FNM, Pereira de Sá VG, Brigatti E. Entropy estimation in bidimensional sequences. Phys Rev E. 2022;105(5–1):054116. doi: 10.1103/PhysRevE.105.054116 35706216

[37] Getis A, Ord JK. The Analysis of Spatial Association by Use of Distance Statistics. Geograph Analys. 1992;24(3):189–206. doi: 10.1111/j.1538-4632.1992.tb00261.x

[38] de Geografia e Estatística IB. Censo demográfico 2010: características da população e dos domicílios: resultados do universo. Rio de Janeiro: IBGE; 2010.

[39] Signorell A. DescTools: Tools for Descriptive Statistics. R package version 0.99.59.7. 2025. Available from: https://cran.r-project.org/package=DescTools

[40] Frank AI. Using measures of spatial autocorrelation to describe socio-economic and racial residential patterns in us urban areas. Socio-economic applications of geographic information science. London: Taylor & Francis; 2003. p. 147–62.

[41] Bivand RS, Wong DWS. Comparing implementations of global and local indicators of spatial association. TEST. 2018;27(3):716–48. doi: 10.1007/s11749-018-0599-x

[42] Bivand R, Krainski E, Piras G. spdep: Spatial Dependence: Weighting Schemes, Statistics and Models. R package version 1.3-3. 2024. Available from: https://cran.r-project.org

[43] Prener C. Areal-weighted interpolation areal R package vignette. 2022. Available from: https://cran.r-project.org/web/packages/areal/vignettes/areal-weighted-interpolation.html

