# Supplementary material for: Is segregation encoded in urban form? An entropy-based analysis
Source: PLoS One. 2026 Oct 23;21(10):e0360856. doi: 10.1371/journal.pone.0360856 (PMC13693912; doi:10.1371/journal.pone.0360856)
Supplement: Supplementary file 1 [file pone.0360856.s001.pdf]

# Is segregation encoded in urban form?
# An entropy-based analysis

## Supplementary Information

Supplementary information is available for the paper “Is segregation encoded in urban form? An entropy-based analysis”. It includes an additional literature review, extended statistical results, grid-level measures of built-form entropy and residential segregation indicators, full piecewise regression results for the built-form entropy–Gini relationship in São Paulo, contextual notes on high- and low-entropy clusters, and supporting figures for the main manuscript.

### Literature review

**Residential segregation and urban form**
Residential segregation studies are usually based on the uneven locational distribution of homogeneous social groups across spatial areas, regions or places. The spatial distribution of social groups as a research subject may be safely dated back at least to the end of the 19th century, namely Charles Booth's poverty maps in London (1889 – Fig. S1b) and Emily Balch's (1895) review of the Hull Maps and Papers in Chicago. An explicit spatial model of social distribution was notably proposed by Burgess’ (1928) Chicago School approach (Fig. S1c) – with remarkable similarities to the national context where it was proposed, in the US (Fig. S1d). Since then, the number of scientific papers explicitly addressing residential segregation has increased exponentially over time (Fig. S1a).

Schelling (1971) proposed a pioneering dynamic model of the evolution of residential segregation based on different ranges of social homophily and the unintended large-scale consequences from individual intended preferences. Resulting residential patterns frequentlhy display segregation levels more than proportional to the level of neighourhood homophily level intended by individuals – e.g. from a random distribution of groups (e.g. blues and reds) over a territory to a distribution able to satisfy a non-segregative neighbourhood homophily from having a minimum 25% of similar neighbours (resulting Moran's I = 0.17) to a segregative preference of having 75% similar neighbours (resulting Moran's I = 0.98). Schelling showed that the only way to satisfy a generalised individual preference at this level is through a highly segregated spatial pattern (Fig. S1e). Except for Schelling's model, which only accounts for adjacency with no real spatial structure taken into account, the morphologies latent in such models and empirical findings are illustrative of different spatial conditions seen as taking part of the materialization of segregation in residential form – from *axial* distributions of income groups along main streets or in *interstitial areas* between them in Booth's London (e.g. the middle class marked in red frequently adjacent to poor or very poor residents in light and dark blue; see Vaughan, 2018) to a *distance-based ringy distribution* in Burgess’ model and in NYC's actual residential patterns.

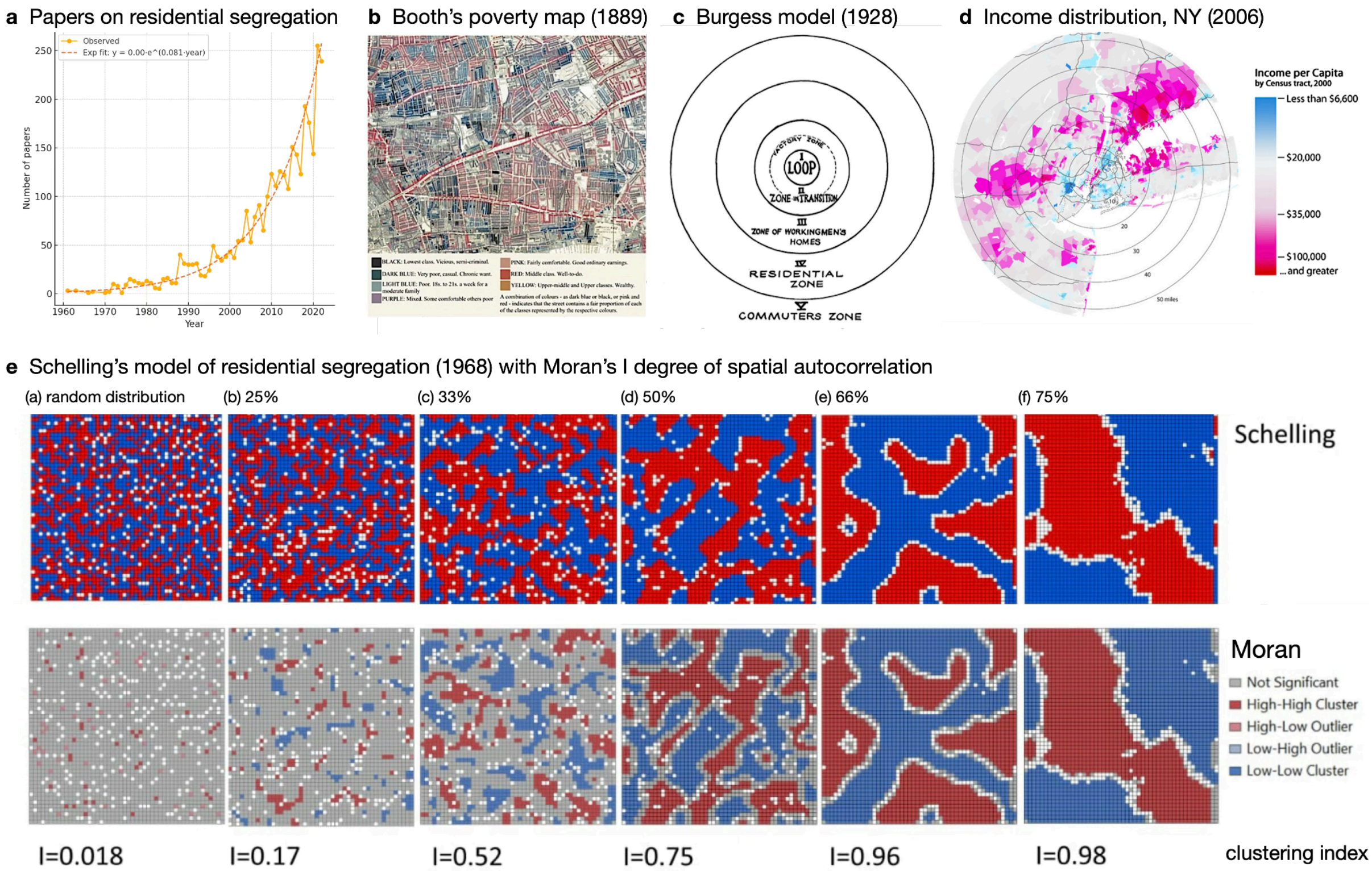


Figure S1 – Approaches to residential segregation: (a) Aggregated number of papers on residential segregation by year in the Scopus dataset, with exponential fit (1960-2022); (b) Charles Booth's pioneering map of poverty in London (1889) associates residential locations with a rough definition of income-based groups; (c) Burgess's (1928) ecological model of residential zones naturalized residential segregation per occupational roles; (d) "City income donuts" showing the residential distribuition of income per capita in the New York and New Jersey state regions (source: US Census 2000/Radical Cartography, 2025), showing the trend of richer residents to locate either in the regional centre that is Manhattan or in the external 'residential zones', and the poor in between – echoing Burgess's model. (e) Schelling's (1971) dynamic model of residential segregation patterns as unintended consequences of non-necessarily segregative individual behaviours. The results of segregation patterns were assessed by applying Moran's I degree of spatial autocorrelation, supporting Schelling's counterintuitive hypothesis.

**From aspatial to spatial measures of residential segregation**

Since Schelling, more works have approached residential segregation as locational patterns, including a role for proximity. A tradition since Duncan and Duncan (1955) established the dissimilarity index as a measure of *evenness* (or group-share) in residential distributions. Other indices of group counts include the Gini segregation index, a Lorenz curve-based evenness measure (James and Taeuber, 1985), and Information-theory or entropy index H (Theil and Finezza, 1971), which extends evenness to multiple groups by comparing each unit's $K$-group entropy to citywide entropy, i.e. the entropy index $H$ measures evenness in residential location based on a weighted average deviation of the entropy in each spatially delimited area (e.g. census tract) from the entropy level of the city as a whole.

These indices are essentially aspatial, working only with the distribution of people limited within areal unit boundaries. In turn, the Spatial Proximity index (SP) (White, 1983) measures clustering, i.e. whether same-group members live closer together than random, using distance-decay, buffer variants (Wong, 1993; Dawkings, 2006) or population-weighted distances between all pairs of individuals in different areal units (Massey et al., 1996).

Massey and Denton (1988) proposed five spatial dimensions of residential segregation: *evenness* (a measure of how evenly groups spread across units or parcels), *exposure* (sharing neighbourhood borders), *concentration* (minorities occupy a share of an area), *clustering* (how close or contiguous are minority units), and *centralisation* (how near minorities live to the city centre, using distance to CBD). Reardon and O'Sullivan (2004) offered a more structured and less internally redundant framework along two axes of distribution: from *evenness* to *clustering*, and from *exposure* (differential proximity between groups) to *isolation* (poor adjacency with members from one's own group). They reframed measures of residential segregation as a scale-dependent spatial process and supplied a toolkit for converting classic aspatial indices into spatial ones, thereby beginning to close the conceptual gap between segregation measures and the morphology of cities.

From the early 1990s forward, scholars attempted to add *contiguity*, *shared borders* or *distance*, progressively weaving spatial aspects into segregation metrics. *Boundary-modified* Dissimilarity (Morrill, 1991) weights each areal unit by the length of shared borders with unlike units. *Weight-modified* Dissimilarity indices (Wong, 1993, 2005) replace each unit's counts with a composite population that blends neighbours within a chosen distance, generating a segregation profile across scales, capturing how segregation contracts or dilates based on an inverse distance weight function to model (potential) interaction intensity. Dawkins (2006) introduces Spatial Gini indices – a *nearest-neighbour* and a monocentric version – that reorder census tracts to reflect which tracts are nearest to each other and which tracts are closest to the central business district, and capture aspects of clustering and centralisation overlooked by aspatial indices.

Such indices are mostly indifferent to urban form or actual locational distributions. Despite their importance in segregation analysis, the foundation of areal units is problematic, since they are, to a large extent, arbitrary definitions subject to the modifiable areal unit problem (MAUP – see Openshaw, 1984). Established in census geography, the areal units of census tracts are artificial constructs for which social data is collected and made available. Their size, shape, or contiguity might bear some relation to actual spatial structures such as urban blocks, but their spatial implications for segregation require careful consideration. For instance, the use of areal units' shared borders as a means to measure exposure brings oversimplifications. Census tracts are, in fact, permeable and continuous in the urban fabric of residential locations and open spaces surrounding them. Shared boundaries between areal units with internal proportions of social groups and distance-based location patterns tell virtually nothing about the actual residential distributions and interfaces between social groups along the permeable structure of open spaces between urban blocks where people live. Any implications about 'exposure' or 'interaction' should be understood as *proxies*, i.e. spatial conditions for *potential* exposure or contact via shared areal unit or boundary, as such conditions do not necessarily lead to actual group contact and interaction. Notwithstanding, such basic spatial *conditions* or *potentials* for contact are frequently conflated with *actual* contact in these approaches, along with sociologically distinct phenomena such as 'contact' and 'interaction', oversimplifying the complexity of social interaction and its material conditions (see Goffman, 1963; Netto et al., 2015). Hence, areal units defined in census geography likely tell very little about the role of urban form in residential segregation.

**A role for urban form in residential segregation?**

More explicit spatial dimensions of residential segregation and roles of the built environment and urban form in restricting or affording potential intergroup contact have been explored in the literature. Approaches to urban form can be generally divided into two components: the intuitively visible built form (BF), i.e. the shape, position and distribution of buildings in the urban environment; and the system of open spaces between buildings as ‘connective voids’ linking them, which has been analytically approached as street networks. We begin with street networks (SN). Space syntax predicts *spatial* segregation as less accessible, topologically deeper positions in SNs, which in turn correlate with potential *social* segregation in the form of lower intensities of co-presence, hence more control over encounters and less potential contact between different social groups or categories, such as inhabitants and strangers (Hillier and Hanson, 1984). Spatial segregation in SNs was then connected with residential segregation – e.g. the clusterisation in “ghettos” of immigrants or the poor – with a high resolution degree (Vaughan, 2007). These innovative approaches still do not precisely estimate the effect of spatial segregation in the street networks on the social segregation between groups, or the role of residential segregation.

Street networks were considered a feature affecting residential segregation, as racially-homogeneous communities were identified in areas internally connected by pedestrian streets, while more poorly connected by non-pedestrian streets of three metropolitan areas in the US (Grannis, 2005). Street connectivity and structure were incorporated as distances between locations of distinct racial groups over road segments and nodes, using SN-distance versions of spatial proximity (Roberto, 2018). Increases in length within SNs due to intricacies and barriers are correlated as deviations from the shortest Euclidean distances as indicators of a role in identified edges between socially homogeneous areas, identified through a divergence index. Physical barriers are identified as perceived by inhabitants as markers of social boundaries between ethnoracial groups (Roberto and Korver-Glenn, 2021). Knaap and Rey (2024) recognise aspects of spatial segregation as a feature in residential segregation, and they also measure distance within SNs based on connectivity and circuity as differences from Euclidean distances. Another measure analyses spatial segregation as topological distances between residential positions of social groups over the street network and all other segments through betweenness and closeness centralities (Saboya and Peres, 2024). Assigning weights to the origin segment based on the number of located households, it calculates the proportions of centrality per group in each street segment as a measure ranging from potential exposure (groups’ centralities equally distributed over segments in the SN) to potential segregation (high topological centrality of one group over others in SN segments). It is a hybrid measure bridging spatial segregation (i.e. how topologically segregated groups are) and a residentially-weighted latent segregation between groups along the street network.

In turn, the role of built form in residential segregation research is more scarcely researched. Approaches have dealt with the role of densities, built form typologies, building footprint patterns and micro-residential segregation in urban blocks and buildings. Relations between morphometric indicators of building footprints, such as density, compactness, distance between buildings and angular variation (‘angle entropy’), were used to generate a typology of neighbourhoods for five metropolitan regions in the US. Size, shape and placement of buildings showed weak statistical associations with socioeconomic characteristics of neighbourhoods, such as income and race (Durst et al., 2024). Other findings also seem to

have found subtle associations between urban form properties of patches of uninterrupted areas of urban development, e.g. patch density, size and shape, as measures of fragmentation, and residential segregation measures spatial evenness and exposure in 84 Colombian cities (Useche et al., 2024). Types of buildings and urban blocks, featuring differences in density, height variance and compactness, were found to be associated with micro-residential segregation of immigrants in Barcelona, Spain, inferred from residential proximity between ethnic groups and measured as within-block dissimilarity (Salazar Miranda, 2020). Other spatial forms of micro-residential segregation can be found as 'vertical residential segregation' – e.g. the distribution of ethnic groups along building floors in Athens, Greece (Maloutas et al., 2001; 2016) and in vertical favelas in Rio de Janeiro, Brazil, where the poorer and black population are likely to live in higher topographies (Netto et al., 2022).

## Results

### BFE and RS-related values per grid cell

| Cell ID | h_c | per capita income | log_income | morans_I | gini |
|---|---|---|---|---|---|
| **1** | 0,310 | 472,05 | 6,157 | 0,260 | 0,157 |
| **2** | 0,314 | 587,10 | 6,375 | 0,340 | 0,204 |
| **3** | 0,374 | 476,74 | 6,167 | 0,220 | 0,157 |
| **4** | 0,362 | 530,80 | 6,274 | 0,220 | 0,168 |
| **5** | 0,329 | 1387,97 | 7,236 | 0,050 | 0,311 |
| **6** | 0,369 | 626,94 | 6,441 | 0,510 | 0,294 |
| **7** | 0,353 | 503,93 | 6,222 | 0,220 | 0,232 |
| **8** | 0,362 | 701,35 | 6,553 | 0,310 | 0,281 |
| **9** | 0,296 | 826,21 | 6,717 | 0,330 | 0,337 |
| **10** | 0,393 | 2788,90 | 7,933 | 0,450 | 0,270 |
| **11** | 0,390 | 1382,53 | 7,232 | 0,560 | 0,405 |
| **12** | 0,388 | 977,91 | 6,885 | 0,640 | 0,505 |
| **13** | 0,389 | 3438,34 | 8,143 | 0,140 | 0,236 |
| **14** | 0,376 | 2545,23 | 7,842 | 0,570 | 0,357 |
| **15** | 0,364 | 1835,67 | 7,515 | 0,420 | 0,275 |
| **16** | 0,330 | 996,85 | 6,905 | 0,390 | 0,289 |
| **17** | 0,416 | 1871,69 | 7,535 | 0,480 | 0,409 |
| **18** | 0,400 | 3914,82 | 8,273 | 0,280 | 0,347 |
| **19** | 0,375 | 4624,58 | 8,439 | 0,270 | 0,192 |
| **20** | 0,334 | 3006,92 | 8,009 | 0,220 | 0,227 |
| **21** | 0,328 | 1255,56 | 7,135 | 0,470 | 0,337 |
| **22** | 0,344 | 588,66 | 6,378 | 0,290 | 0,183 |
| **23** | 0,314 | 534,64 | 6,282 | 0,100 | 0,162 |
| **24** | 0,393 | 1377,14 | 7,228 | 0,270 | 0,366 |
| **25** | 0,405 | 2478,82 | 7,816 | 0,550 | 0,265 |
| **26** | 0,370 | 4372,02 | 8,383 | 0,350 | 0,260 |
| **27** | 0,383 | 4899,97 | 8,497 | 0,020 | 0,158 |
| **28** | 0,366 | 3237,60 | 8,083 | 0,340 | 0,238 |

| | | | | | |
|---|---|---|---|---|---|
| **29** | 0,280 | 1858,10 | 7,527 | 0,220 | 0,244 |
| **30** | 0,342 | 1436,44 | 7,270 | 0,370 | 0,283 |
| **31** | 0,360 | 976,56 | 6,884 | 0,230 | 0,188 |
| **32** | 0,331 | 710,75 | 6,566 | 0,220 | 0,205 |
| **33** | 0,391 | 412,11 | 6,021 | 0,180 | 0,170 |
| **34** | 0,387 | 1517,14 | 7,325 | 0,580 | 0,376 |
| **35** | 0,392 | 3262,74 | 8,090 | 0,210 | 0,208 |
| **36** | 0,363 | 3695,34 | 8,215 | 0,290 | 0,221 |
| **37** | 0,248 | 1484,22 | 7,303 | 0,360 | 0,259 |
| **38** | 0,276 | 1494,47 | 7,310 | 0,240 | 0,288 |
| **39** | 0,324 | 2110,58 | 7,655 | 0,240 | 0,254 |
| **40** | 0,335 | 1510,34 | 7,320 | 0,410 | 0,264 |
| **41** | 0,340 | 898,09 | 6,800 | 0,210 | 0,172 |
| **42** | 0,327 | 697,65 | 6,548 | 0,240 | 0,157 |
| **43** | 0,336 | 701,86 | 6,554 | 0,060 | 0,181 |
| **44** | 0,365 | 585,59 | 6,373 | 0,430 | 0,163 |
| **45** | 0,332 | 3122,24 | 8,046 | 0,270 | 0,283 |
| **46** | 0,277 | 2786,77 | 7,933 | 0,320 | 0,265 |
| **47** | 0,296 | 2286,56 | 7,735 | 0,460 | 0,293 |
| **48** | 0,296 | 1018,16 | 6,926 | 0,200 | 0,274 |
| **49** | 0,271 | 1079,72 | 6,984 | 0,080 | 0,246 |
| **50** | 0,287 | 1127,69 | 7,028 | 0,480 | 0,363 |
| **51** | 0,328 | 1075,60 | 6,981 | 0,390 | 0,238 |
| **52** | 0,352 | 1035,66 | 6,943 | 0,260 | 0,179 |
| **53** | 0,348 | 746,64 | 6,616 | 0,160 | 0,151 |
| **54** | 0,335 | 573,74 | 6,352 | 0,300 | 0,198 |
| **55** | 0,317 | 493,77 | 6,202 | 0,200 | 0,159 |
| **56** | 0,331 | 405,08 | 6,004 | 0,160 | 0,121 |
| **57** | 0,377 | 894,46 | 6,796 | 0,380 | 0,242 |
| **58** | 0,387 | 1238,76 | 7,122 | 0,280 | 0,238 |
| **59** | 0,372 | 1109,37 | 7,012 | 0,320 | 0,232 |
| **60** | 0,401 | 963,58 | 6,871 | 0,280 | 0,259 |
| **61** | 0,376 | 2006,27 | 7,604 | 0,330 | 0,244 |
| **62** | 0,387 | 1305,67 | 7,174 | 0,460 | 0,251 |
| **63** | 0,339 | 777,39 | 6,656 | 0,240 | 0,167 |
| **64** | 0,344 | 652,13 | 6,480 | 0,290 | 0,195 |
| **65** | 0,331 | 555,93 | 6,321 | 0,460 | 0,234 |
| **66** | 0,268 | 583,11 | 6,368 | 0,340 | 0,186 |
| **67** | 0,294 | 470,12 | 6,153 | 0,350 | 0,171 |
| **68** | 0,344 | 561,35 | 6,330 | 0,400 | 0,203 |
| **69** | 0,345 | 697,92 | 6,548 | 0,360 | 0,241 |
| **70** | 0,371 | 1315,26 | 7,182 | 0,450 | 0,213 |
| **71** | 0,326 | 531,52 | 6,276 | 0,530 | 0,244 |

Table S1 – Metrics for São Paulo’s 3×3 km grid cells: built-form entropy ($h_c$), average per-capita income, log(income), Moran’s I (local clustering), and Gini (within-cell inequality).

**Built-form entropy vs. Gini**

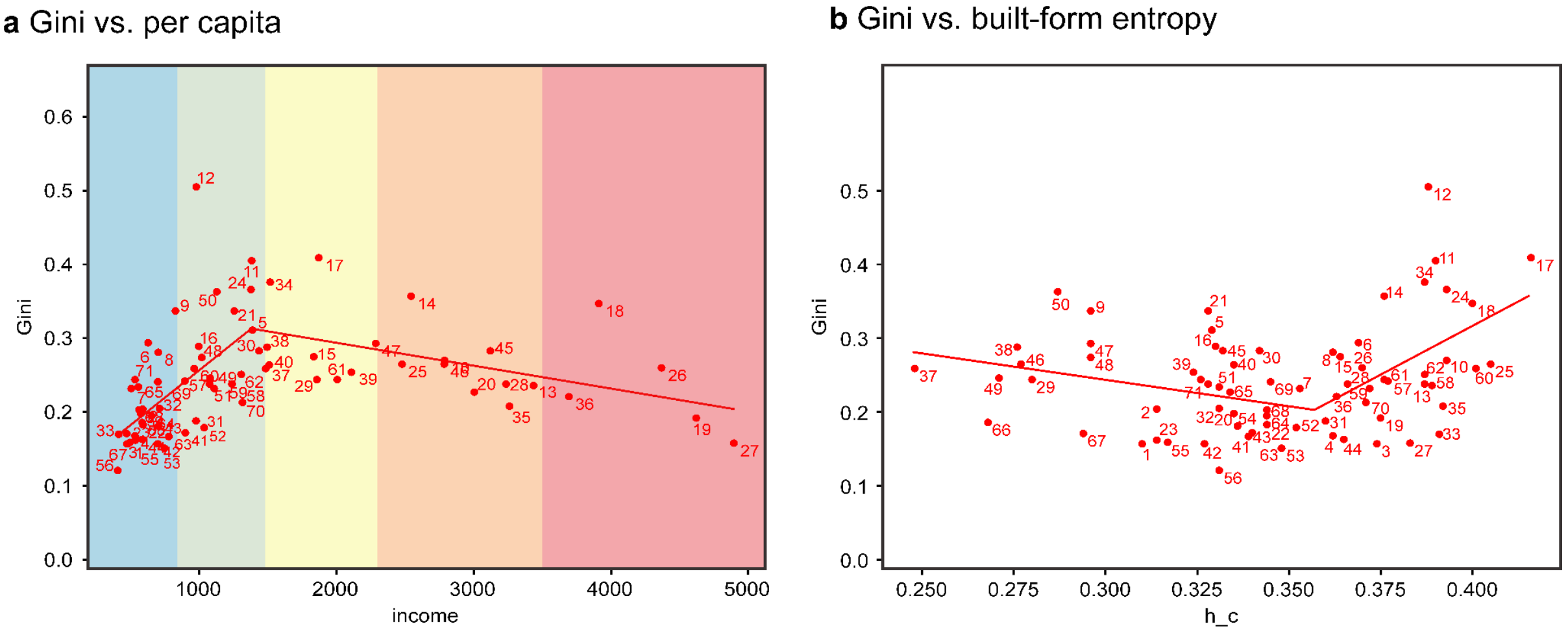


Figure S2 – Gini versus built-form entropy; solid line, piecewise regression.

## Discussion

**Contextual notes on selected high- and low-entropy clusters**

To complement the discussion in the main manuscript, this section provides brief historical and morphological descriptions of selected cells drawn from the high- and low-entropy clusters. These contextual notes help situate the examples used in Fig.6 within the broader urban development of São Paulo, and clarify how distinct local histories may underlie the socio-spatial configurations associated with similar built-form entropy values. Computed within the 3×3 km cells, Gini and Moran's I are used to capture internal socio-spatial composition across different empirical situations:

**High built-form entropy cluster**

The high built-form entropy cluster lies southwest of São Paulo’s historic CBD (see Fig. 2b–c in the main manuscript). It includes areas shaped by different urbanisation trajectories, but sharing morphologically intricate fabrics.

Jaguaré (cell 34) and Rio Pequeno (cell 24), in the West Zone, were mainly urbanised from the 1960s onward, initially by factory and construction workers, including informal settlements. Rio Pequeno later underwent residential verticalisation as improved connectivity attracted middle- and upper-middle-class developments. The district now combines favelas and middle-income areas, forming small clusters of lower- to middle-income groups in the north and higher-income groups in the south. Jaguaré’s urban blocks were designed in a labyrinthine pattern in 1935. Butantã (cell 25) also mixes income groups, mainly middle-income households with higher-income pockets in its southern area.

Closer to the CBD are some of São Paulo’s highest-income areas. Pinheiros and Perdizes (cell 35; see Fig. 6 in the main manuscript), urbanised along streetcar and railway lines in the nineteenth century, evolved from a mixed industrial–residential zone into a gentrified high-income district. Jardim Paulista (cell 27; see Fig. 5 in the main manuscript), structured by major avenues such as Paulista and Rebouças, combines rectilinear and organic street networks planned since the 1910s. It is now heavily verticalised and homogeneously

high-income, with no visible internal clusters and the lowest Moran’s I value in São Paulo (0.020).

Cell 17, comprising Vila Sonia and Morumbi, combines two socially unequal districts. It records the second-highest Gini value (0.409) and the ninth-highest Moran’s I (0.480). Vila Sonia is predominantly middle- to lower-income, with small pockets of affluence along its eastern border with Morumbi. Morumbi, by contrast, developed into low-density, winding subdivisions dominated by detached houses and gated condominiums, and has the highest average per-capita income in the city. The juxtaposition of the city’s wealthiest district with marked income inequality and strong local clustering yields the highest built-form entropy in São Paulo (($h_c \approx 0.416$)).

Itaim Bibi (cell 18), parcelled from rural properties in the nineteenth century and densified after the 1950s, became a prime business and luxury-residential district, including along Faria Lima Avenue, with dispersed high-rise clusters of upper-middle- and high-income dwellings. Moema (cell 19) began urbanising in 1913, with rapid growth after the 1970s through rectilinear blocks and detached vertical buildings around Parque do Ibirapuera. It is now a socially homogeneous area with the city’s second-highest per-capita income and highest HDI. Finally, Santo Amaro (cells 13 and 10), formerly an independent municipality incorporated into São Paulo in 1935, retains its own sub-centre linking the southern expansion, with upper-middle-income housing along main corridors and lower-income pockets in its periphery.

Taken together, these high-entropy cells show that similar entropy signatures may emerge from distinct urban processes, including affluent enclaving, mixed-income differentiation, and incremental or partially informal urbanisation.

**Low built-form entropy cluster: historic CBD and eastern expansion**

The larger low built-form entropy cluster includes the historic CBD and its eastern expansion, among the most spatially ordered areas of the city. These areas were largely urbanised before the recent wave of typological replacement by detached vertical buildings.

República and Sé (cell 37; see Fig. 6 in the main manuscript) comprise the historic CBD: a dense vertical core of multi-storey commercial and residential blocks with street-aligned façades and minimal setbacks, producing a highly consistent built fabric and the lowest built-form entropy in the city ($h_c \approx 0.248$). Since the 1960s, as elites moved out, the area developed marked income heterogeneity, with lower-income residents concentrated in older housing to the east and higher-income users to the west, tied to commercial activities. This produces internal disparities (Gini = 0.259) and moderate local clustering (Moran’s I = 0.360).

Just east of the CBD, Brás (the western part of cell 38) presents a dense small-block grid originating in early industrial expansion, with narrow plots, continuous façades, and mixed wholesale, storage, and residential uses. Many former industrial buildings have been converted to retail or services, preserving strong ground-floor continuity. Per-capita incomes remain predominantly low. Belém (the northern part of cell 38) maintains rectilinear blocks and aligned façades, with former industrial buildings converted into lofts or commercial uses

and with pockets of low-rise housing and small businesses. Per-capita incomes are in the lower-middle range.

Mooca (the southern part of cell 38) and Ipiranga (cell 29) retain traces of their industrial past, including factory blocks and workers' housing, now blended with newer residential buildings under progressive densification. Their urban fabric remains relatively regular and continuous, despite moderate piecemeal verticalisation. Per-capita incomes are in the middle- to upper-middle-income range. Vila Guilherme and surrounding areas (cell 49) show a mostly regular lotting pattern, with denser low-rise fabric near the Tietê plain and growing verticalisation along main arteries. Per-capita incomes are largely within lower- to middle-income ranges, reflecting a modest and mixed socio-economic profile.

Northeast of the CBD, Bom Retiro and Pari (cells 48–49) combine rectilinear grids with compact typologies and continuous façades in former industrial and warehouse buildings, now interspersed with emerging residential uses and relatively few high towers. Income levels remain modest, dominated by lower-income groups. Further east, Vila Maria and Tatuapé (cell 50) evolved from modest single-family houses into compact low-rise blocks with continuous building frontages arranged in orthogonal and radial block systems. Most households are now in middle- to upper-middle-income brackets, reflecting the area's consolidation as a service-oriented residential district.

These low-entropy areas illustrate how ordered fabrics may still coincide with internal socio-economic differentiation and moderate clustering, especially in central areas shaped by older planning regimes, industrial legacies, and subsequent selective occupation.

**Smaller low-entropy cluster in the eastern periphery**

A smaller low-entropy cluster appears in São Paulo's far eastern periphery, an area of late and uneven urbanisation long shaped by infrastructure deficits, distance from central hubs, and socio-economic marginalisation.

São Miguel, Jardim Helena, Vila Curuçá, and Itaim Paulista (cells 66–67; see Fig. 6 in the main manuscript) developed through the eastward expansion of the metropolis and the later addition of regional train stations. Their urban fabric combines older low-density housing, informal settlements, compact residential blocks, and linear commercial corridors. Closer to rail lines and main arteries, more regular grids and mixed-use structures appear. These districts occupy the lower tiers of the city's income hierarchy and are characterised by modest to low per-capita incomes.

This peripheral low-entropy cluster differs from the central low-entropy cluster in its socio-economic position and development history, but shares with it a comparatively ordered urban fabric. In this sense, low built-form entropy in São Paulo is not limited to affluent or historic central areas, but may also characterise peripheral districts shaped by simpler and more repetitive urbanisation patterns.

## Method

We used the average per-capita income reported for each tract as our socioeconomic indicator. Figure S3 illustrates how census tracts relate to a single 3×3 km grid cell in our analysis. We also calculated the Gini index for each grid cell.

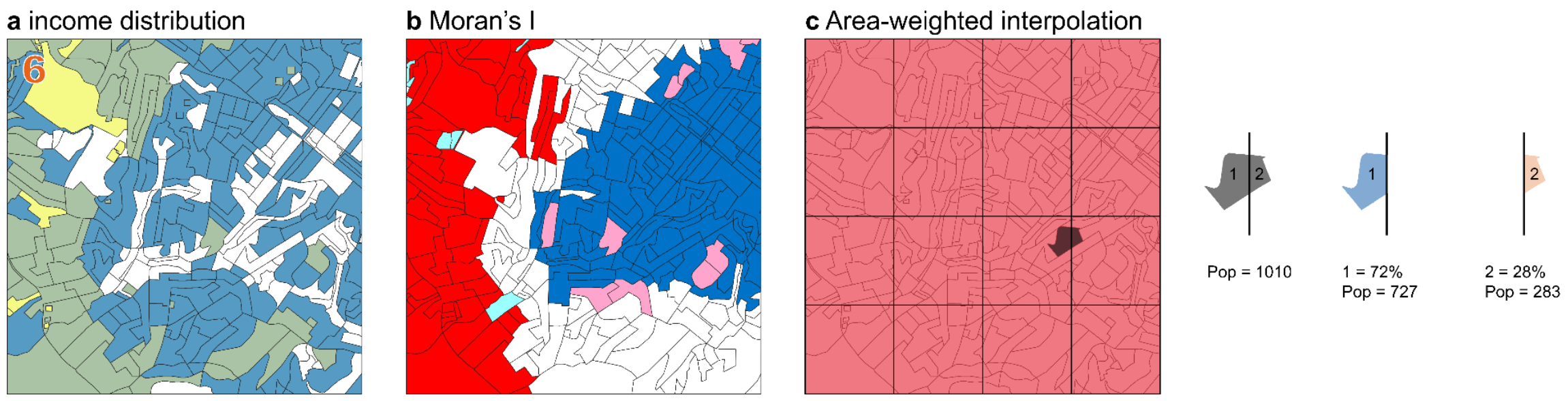


Figure S3 – Steps in analysing residential segregation based on income (per capita) in São Paulo: (a) income distribution in census tracts; (b) Moran's I applied to a single 3x3km cell; (c) area-weighted interpolation in the transfer of attributes from census tracts to the grid of 3 x 3km cells. Note that tract scale is remarkably smaller than the cell, allowing for a robust estimation of the Gini and Moran's .

## References for Supplementary Information


Balch, E. G. H. *Hull House Maps and Papers* (T. Y. Crowell, 1895).

Booth, C. *Poverty Maps of London* (British Library, 1889).

Burgess, E. W. Residential segregation in American cities. *Ann. Am. Acad. Polit. Soc. Sci.* **140**, 105–115 (1928).

Dawkins, C. The spatial pattern of black-white segregation in US metropolitan areas: an exploratory analysis. *Urban Stud.* **43**, 1943–1969 (2006).

Duncan, O. D. & Duncan, B. A. Methodological analysis of segregation indexes. *Am. Sociol. Rev.* **20**, 210–217 (1955).

Durst, N. J., Sullivan, E. & Jochem, W. C. The spatial and social correlates of neighborhood morphology: evidence from building footprints in five US metropolitan areas. *PLoS ONE* **19**, e0299713 (2024).

Goffman, E. *Behaviour in Public Spaces* (Free Press, 1963).

Grannis, R. T-communities: pedestrian street networks and residential segregation in Chicago, Los Angeles, and New York. *City Community* **4**, 295–321 (2005).

Hillier, B. & Hanson, J. *The Social Logic of Space* (Cambridge Univ. Press, 1984).

James, D. R. & Taeuber, K. E. Measures of segregation. *Sociol. Methodol.* **15**, 1–32 (1985).

Knaap, E. & Rey, S. Segregated by design? Street network topological structure and the measurement of urban segregation. *Environ. Plan. B Urban Anal. City Sci.* **51**, 1408–1429 (2024).

Massey, D. S. & Denton, N. A. The dimensions of residential segregation. *Soc. Forces* **67**, 281–315 (1988).

Massey, D. S., White, M. J. & Phua, V. C. The dimensions of segregation revisited. *Sociol. Methods Res.* **25**, 172–206 (1996).

Miranda, A. S. The shape of segregation: the role of urban form in immigrant assimilation. *Cities* **106**, 102852 (2020).

Morrill, R. On the measure of geographic segregation. *Geogr. Res. Forum* **11**, 25–36 (1991).

Netto, V. M., Soares, M. P. & Paschoalino, R. Segregated networks in the city. *Int. J. Urban Reg. Res.* **39**, 1084–1102 (2015).

Netto, V. M., Carvalho, C., Fiszon, M. & Couto, Y. Micro-segregation in Rio de Janeiro. In *Vertical Cities* 154–171 (Edward Elgar Publishing, 2022).

Openshaw, S. Ecological fallacies and the analysis of areal census data. *Environ. Plan. A* **16**, 17–31 (1984).

Reardon, S. F. & O'Sullivan, D. Measures of spatial segregation. *Sociol. Methodol.* **34**, 121–162 (2004).

Roberto, E. The spatial proximity and connectivity method for measuring and analyzing residential segregation. *Sociol. Methodol.* **48**, 182–224 (2018).

Saboya, R. T. & Peres, O. M. Chasm: a configurational measure of socio-spatial residential segregation. *Environ. Plan. B Urban Anal. City Sci.* **52**, 1464–1481 (2025).

Schelling, T. C. Dynamic models of segregation. *J. Math. Sociol.* **1**, 143–186 (1971).

Theil, H. & Finizza, A. J. A note on the measurement of racial integration of schools by means of informational concepts. *J. Math. Sociol.* **1**, 187–193 (1971).

Useche, A. F., Sarmiento, O. L., Alvarez-Rivadulla, M. J., Medina, P., Higuera-Mendieta, D. & Montes, F. Spatial segregation patterns and association with built environment features in Colombian cities. *Cities* **152**, 105217 (2024).

Vaughan, L. The spatial syntax of urban segregation. *Prog. Plann.* **67**, 199–294 (2007).

Vaughan, L. *Mapping Society: The Spatial Dimensions of Social Cartography* (UCL Press, 2018).

White, M. J. The measurement of spatial segregation. *Am. J. Sociol.* **88**, 1008–1018 (1983).

Wong, D. S. Spatial indices of segregation. *Urban Stud.* **30**, 559–572 (1993).

Wong, D. W. Formulating a general spatial segregation measure. *Prof. Geogr.* **57**, 285–294 (2005).
